# Engineering global transcription to tune lipophilic properties in *Yarrowia lipolytica*

**DOI:** 10.1186/s13068-018-1114-z

**Published:** 2018-04-19

**Authors:** Man Wang, Guan-Nan Liu, Hong Liu, Lu Zhang, Bing-Zhi Li, Xia Li, Duo Liu, Ying-Jin Yuan

**Affiliations:** 1 Key Laboratory of Systems Bioengineering (Ministry of Education), School of Chemical Engineering and Technology, Tianjin University, Tianjin, 300072 People’s Republic of China; 2 SynBio Research Platform, Collaborative Innovation Center of Chemical Science and Engineering (Tianjin), Tianjin University, Tianjin, 300072 People’s Republic of China; 3 College of Biotechnology and Pharmaceutical Engineering, Nanjing Tech University, Nanjing, 211816 People’s Republic of China

**Keywords:** *Yarrowia lipolytica*, Global transcription engineering, DNA assembly, Transcription factor, Beta-carotene, Fatty acid, Lipid

## Abstract

**Background:**

Evolution of complex phenotypes in cells requires simultaneously tuning expression of large amounts of genes, which can be achieved by reprograming global transcription. Lipophilicity is an important complex trait in oleaginous yeast *Yarrowia lipolytica*. It is necessary to explore the changes of which genes’ expression levels will tune cellular lipophilic properties via the strategy of global transcription engineering.

**Results:**

We achieved a strategy of global transcription engineering in *Y. lipolytica* by modifying the sequences of a key transcriptional factor (TF), *SPT15*-*like* (*Yl*-*SPT15*). The combinatorial mutagenesis of this gene was achieved by DNA assembly of up to five expression cassettes of its error-prone PCR libraries. A heterologous beta-carotene biosynthetic pathway was constructed to research the effects of combined *Yl*-*SPT15* mutants on carotene and lipid production. As a result, we obtained both an “enhanced” strain with 4.7-fold carotene production and a “weakened” strain with 0.13-fold carotene production relative to the initial strain, nearly 40-fold changing range. Genotype verification, comparative transcriptome analysis, and detection of the amounts of total and free fatty acids were made for the selected strains, indicating effective tuning of cells’ lipophilic properties. We exploited the key pathways including RNA polymerase, ketone body metabolism, fatty acid synthesis, and degradation that drastically determined cells’ variable lipophilicity.

**Conclusions:**

We have examined the effects of combinatorial mutagenesis of *Yl*-*SPT15* on cells’ capacity of producing beta-carotene and lipids. The lipophilic properties in *Y. lipolytica* could be effectively tuned by simultaneously regulating genome-wide multi-gene expression levels. The exploited gene targets and pathways could guide design and reconstruction of yeast cells for tunable and optimal production of other lipophilic products.

**Electronic supplementary material:**

The online version of this article (10.1186/s13068-018-1114-z) contains supplementary material, which is available to authorized users.

## Background

Metabolic engineering and synthetic biology has developed versatile tools to engineer certain metabolic pathways to get optimal product synthesis and hyperburst of target functions [1–5]. However, evolution of complex traits in cells always demands arranging the expression levels of larger range of genes. If we can construct a wide change of gene expression, we can also understand why and how cells choose and accept special changes to meet target functions. An accessible strategy is global transcriptional machinery engineering (gTME), which was firstly reported by Stephanopoulos’ group [6, 7]. Gene sequences of transcription factors are subjected to mutation via error-prone polymerase chain reaction (PCR) or site-directed mutagenesis methods, leading to changeable promotor preferences and efficiency of RNA polymerase, which further modulates genome-wide transcription levels to varied extents. The diversity of changed transcriptomes confers diverse probable phenotypes on cells [6–12]. This strategy has already been used in several organisms, such as *Escherichia coli*, *Saccharomyces cerevisiae*, and *Zymomonas mobilis*, offering effective methods to generate novel mutants with enhanced environmental tolerance, metabolite production, and substrate utilization [6–8, 11, 12]. Besides, several transcription factors have been successfully engineered to optimize performance of strains, such as sigma factor, CRP, Spt15, H-NS, and Hha [10, 13–15]. In oleaginous yeast *Yarrowia lipolytica*, a similar strategy of global transcription engineering is in demand to tune cellular complex phenotypes such as lipophilicity which usually requires participation of large amounts of genes.

In recent years, *Y. lipolytica* attracts more attention as this yeast produces high-level lipids and its gene manipulation is available [16–19]. Metabolic pathway engineering has been used to improve production of several valuable products such as biofuels, polyunsaturated fatty acids, and carotenoids [20–25]. The experience obtained from rational engineering of model organism *E. coli* and *S. cerevisiae* has also been proved effective in *Y. lipolytica*, for example, for carotenoid synthesis. Wu et al. [26] improved the production of beta-carotene in *E. coli* to 44.2 mg/g DCW in flasks by combining strategies of modular pathway engineering and membrane engineering. Xie et al. [27] constructed a *S. cerevisiae* strain producing 1.156 g/L (20.79 mg/g DCW) of carotenoids using sequential control strategy in fermentation. Chen et al. [28] got 1.65 g/L (55.56 mg/g DCW) of lycopene from fermentation of *S. cerevisiae* by combining chassis engineering and heterologous pathway engineering. As for *Y. lipolytica*, the native *tHMGR* and *ERG* series genes were overexpressed in Gao’s work to improve fermentative production of beta-carotene to 4 g/L [23]. A recent work reported that the strain highly accumulating lipids could also highly produce carotenoids [29]. They achieved a fermentative production of beta-carotene of 6.5 g/L (90 mg/g DCW) with a concomitant production of 42.6 g/L of lipids. However, the deep tuning mode of competitive synthesis of fatty acids, lipids, and other heterologous lipophilic products like carotenoids was not clearly elucidated. Some distant pathway enzymes such as transporters and coenzymes could also tune lipid product synthesis [21, 30]. It was necessary to research how *Y. lipolytica* tunes its gene expression to affect internal lipophilicity to gain appropriate levels of native lipids and heterologous lipophilic products.

**Fig. 1:**
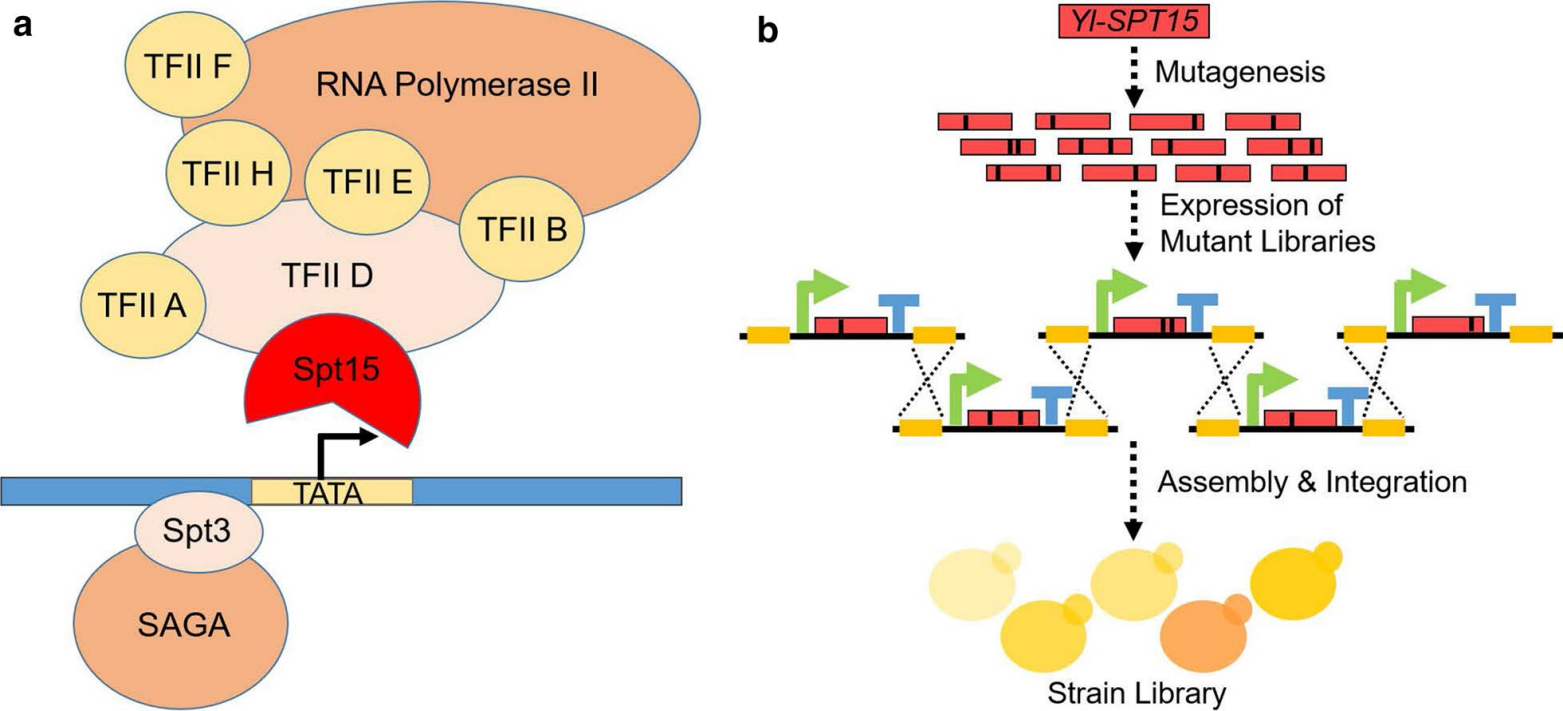
Concept of global transcription engineering in *Y. lipolytica*. **a** Spt15 is a key transcription factor (TF) in TFIID of *S. cerevisiae*. By BLAST analysis, we got a Spt15-like protein named as Yl-Spt15 coded by the gene *YALIOB23056g*. This Yl-Spt15 was probable to take the similar role as Spt15. **b** The manipulated *Yl*-*SPT15* mutant libraries were inserted in five expression cassettes and assembled meanwhile integrated in yeast chromosomal GUT2 site by in vivo homologous recombination. The cells obtaining different mutant combinations were screened and selected

Here we report a strategy of engineering global transcription in *Y. lipolytica* to tune the complex lipophilicity and detailed lipophilic properties such as carotenoids, fatty acids and lipid bodies. In eukaryote’s general polymerase II transcription factor D (TFIID), a central Spt15 factor is TATA-binding protein (TBP), which is able to globally control the transcription of various metabolic and regulatory genes [31]. This factor has four conservative functional regions, namely “repeat element 1,” “helix 2,” “repeat element 2,” and “helix 2’.” Some mutations of Spt15 resulted in pleiotropic changes of gene transcription levels [32, 33]. The *SPT15* gene mutants were demonstrated feasibility to phenotypic evolution in *S. cerevisiae* in many ways, such as enhancing ethanol production [34], improving xylose fermentation [10], and enhancing adaptation to corn cob acid hydrolysate [11]. For modifying Yl-Spt15 (a potential TF) in *Y. lipolytica*, we constructed combinatorial mutagenesis of its coding gene and screened the optimal mutant combinations for enhanced production of beta-carotene, which was synthesized as a non-native product by a heterologous pathway (Fig. 1). A 40-fold changing range of carotene production was obtained and the further analysis of the selected strains indicated effective tuning of lipophilic properties. We exploited the key changed pathways and offered guidance for tuning yeast lipophilicity to produce other high value-added lipophilic products.

## Results

### Expression of *Yl*-*SPT15* mutant libraries in assembled cassettes

According to the amino acid sequence of Spt15 in *S. cerevisiae*, we searched the conserved domains in *Y. lipolytica* by BLAST in NCBI database and got a Spt15-like protein named as Yl-Spt15 coded by *YALIOB23056g* on chromosome B (Fig. 1a and Additional file 1: Figure S1). This Yl-Spt15 owned the same conserved regions of “repeat element 1,” “helix 2,” “repeat element 2,” and “helix 2'” with Spt15 (Additional file 1: Figure S1, Additional file 2). Since the mutants of Spt15 in *S. cerevisiae* were proved effective to promote phenotypic evolution in many ways, we decided to construct the mutants of its potential counterpart Yl-Spt15 in *Y. lipolytica* to test its effects on product synthesis such as lipophilic products [10, 11, 34]. The manipulated *Yl*-*SPT15* mutant libraries (see “Methods” for detailed construction process) were inserted in designed five expression cassettes and assembled meanwhile integrated in yeast chromosomal GUT2 site by in vivo homologous recombination (HR) (Fig. 1b, Additional file 1: Figure S2a, b and “Methods”). The correct rate of three-cassette assembly and integration at GUT2 site was about 7.3% and could be further improved to 18.0% in the strain with *ku70* knockout (Additional file 1: Figure S3). Although the efficiency was not so high, this site primarily could be employed for integration of multiple *Yl*-*SPT15* mutants. This kind of combinatorial mutagenesis of *Yl*-*SPT15* permitted increased frequencies of both wild-type copies and mutant-type copies, offering expanded tuning range to identify dominant mutations that cause new functions in the presence of other unaltered genes.

To test whether the design of combinatorial *Yl*-*SPT15* mutation was effective to tune metabolism of lipophilic products, we constructed a heterologous hybrid pathway to synthesize beta-carotene, a typical lipophilic product. Beta-carotene was used here for its lipophilic trait and visible color convenient for available yeast colony selection [35]. The heterologous carotene would introduce a change in the metabolism and composition of cell’s lipophilic properties, and through selection of drastically changed carotene production in yeast colonies, we obtained a chance to learn how cells tuned both global gene transcription and intrinsic lipophilicity. The carotene pathway containing four modules of H0-EXP1p-*crtE*-XPR2t-H1, H1-TEFp-*crtB*-LIP2t-H2, H2-GPDp-*crtI*-OCTt-H3, H3-GPATp-*crtY*-PEX16t-H4 and a left module “rDNAL-Ura3-H0” and a right module “H4-rDNAR” were assembled and integrated in chromosomal rDNA site (Additional file 1: Figure S2c, the strain was named as Yl_ini). Both “rDNAL” and “rDNAR” were 700 bp referenced to Gao’s design [17]. We got a similar phenomenon that the integration in rDNA was actually not exactly single copy. Quantitative PCR (Q-PCR) indicated that Yl_ini slightly underwent unexpected additional integrations of partial cassettes containing TEFp, GPDp, and GPATp (Additional file 1: Figure S4, Additional file 3: Table S1, “Methods”). However, we considered that these already obtained integrations were stable based on the nature of rDNA site, and the initial carotene production was also stable as detected. Based on this Yl_ini strain, we assembled sequential five cassettes of mutant libraries in yeast cells and successfully selected obvious color-changed yeast colonies. A most “enhanced” colony and a most “weakened” colony was selected by eye judgement for further analysis. It was proved that only cassettes of wild-type *Yl*-*SPT15* did not affect colony’s color and carotene production (Additional file 1: Figure S5a).

### Effects of combinatorial mutagenesis of *Yl*-*SPT15* on beta-carotene production

**Fig. 2:**
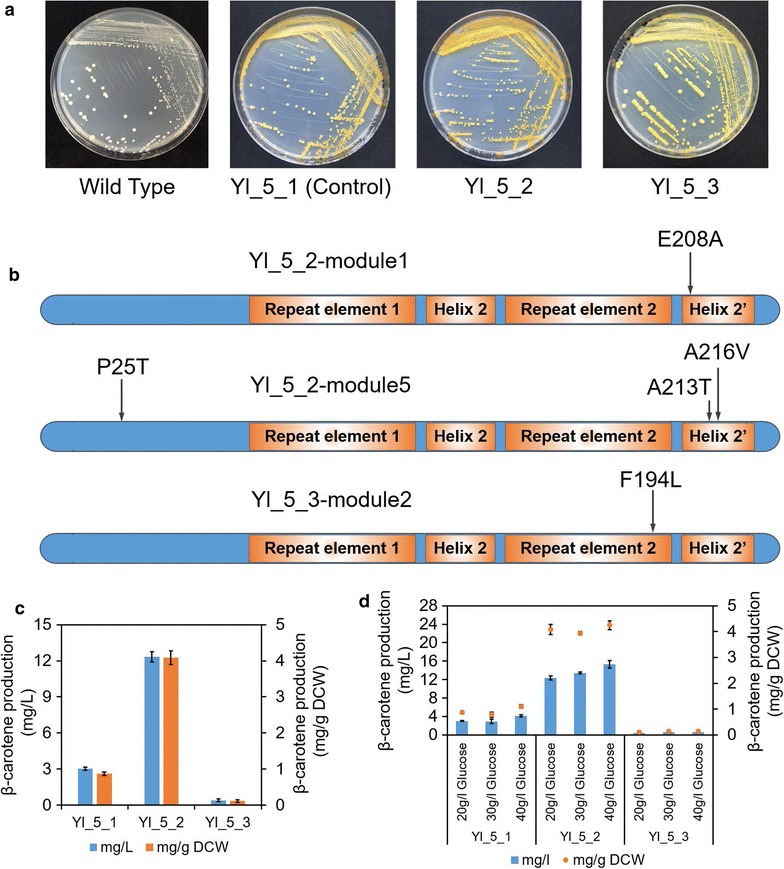
Differential genotypes and phenotypes between selected strains. **a** Compared with the control strain Yl_5_1 containing cassettes of wild-type *Yl*-*SPT15*, a strain Yl_5_2 with deep yellow color and a strain Yl_5_3 with light yellow color were picked for further analysis. **b** The genotypes were tested for the mutants in all five modules contained in the two selected strains. **c** The production of beta-carotene in these strains was tested. **d** The strains were cultivated under 20, 30, and 40 g/L glucose condition to test their production of beta-carotene. All error bars indicate ± standard deviation, *n* = 3

The control strain containing cassettes of wild-type *Yl*-*SPT15* was named as Yl_5_1, the visible “enhanced” strain was named as Yl_5_2, and the visible “weakened” strain was named as Yl_5_3 as follows (Fig. 2a). Firstly, the genotypes of selected strains were tested. According to the results of normal PCR and sequencing with special primers (“Methods” and Additional file 3: Table S2), the left integration site was exactly located upstream of *GUT2*’s open read frame (ORF), but the right integration site was not completely explicit. The designed homologous arms (H8) were 70% in accordance with the sequenced PCR products, but GUT2R could not be verified in alignment. This unexpected integration might lead to partial gene knockdown instead of knockout. The results of Q-PCR proved this speculation, as the expression of *GUT2* was only partially suppressed to nearly half of previous level (Additional file 1: Figure S6 and Additional file 3: Table S1, “Methods”). By contrast, the expression of *Yl*-*SPT15* was almost at the same level across all the tested strains including Yl_ini, Yl_5_1, Yl_5_2, and Yl_5_3, meaning the cells kept the *Yl*-*SPT15*’s transcription at a conservative level, although extra five cassettes were integrated.

The sequencing results showed that Yl_5_2 did receive more than one mutation in different cassettes, one (Glu208Ala) localized in the first module and three (Pro25Thr, Ala213Thr, Ala216Val) localized in the fifth module (Fig. 2b, Additional file 3: Table S3). The Yl_5_3 contained only one mutation (Phe194Leu) localized in the second module. All of the other modules at the expected locations in strain Yl_5_1, Yl_5_2, and Yl_5_3 were the cassettes of wild-type *Yl*-*SPT15*. As seen, four of all five mutations existed in the conserved transient region across “repeat element 2” (beta sheets) to “helix 2’” (alpha helix) [32, 36], indicating that this region was necessary to determine TF’s activity, similar within *S. cerevisiae* [6].

The production of beta-carotene was tested for these three strains (Fig. 2c). We chose defined media instead of rich media to remove interference caused by rich nutritional conditions. The carotene production in Yl_5_1 was 3.03 mg/L (0.87 mg/g DCW). Yl_5_2 got enhanced production as 12.34 mg/L (4.09 mg/g DCW, 4.7-fold of the Yl_5_1’s value), and Yl_5_3 got decreased production as 0.40 mg/L (0.11 mg/g DCW, 0.13-fold of the Yl_5_1’s value). The distinguished productions were directly reflected by the varied depth of strains’ yellow color (Fig. 2a). The growth rate was slightly different between strains (Additional file 1: Figure S5b). The Yl_5_2 with highest carotene production got lowest OD_600_, but the Yl_5_3 with lowest carotene production got highest OD_600_. An evaluation of the effects of each mutation on carotene production was also done. The exact single or combinatorial point mutants were introduced to natural *Yl*-*SPT15* gene by PCR instead of directly cloning the sequencing-verified mutants from genome. Their individual expression cassette on plasmid pLD-EcYl was transformed into Yl_5_1. The carotene detection results suggested that the mutants in module 5 in Yl_5_2 and the mutant in module 2 in Yl_5_3 played significant role of impacting carotene production in each strain. It was also proved that the enhanced phenotype in Yl_5_2 was a result of combined efforts from different mutants (Additional file 1: Figure S7).

To test the influences of different levels of glucose, the strains were cultivated under 20, 30, and 40 g/L glucose keeping other conditions constant (Fig. 2d). Generally, the carotene yields increased as the glucose concentration increased, but there was slight impact on milligram per gram DCW values. When the glucose concentration was 40 g/L, we got the most enhanced production of carotene as 15.29 mg/L (4.29 mg/g DCW) in strain Yl_5_2, a limited 23.96% improvement. It was supposed that *Y. lipolytica* strains under SC-Ura-Leu fermentation could not present highest milligram per liter yield, and the SC other than YPD medium was used here for clearly detecting and analyzing cells’ internal change [37].

### Exploration of key changed genes and pathways by transcriptome analysis

**Fig. 3:**
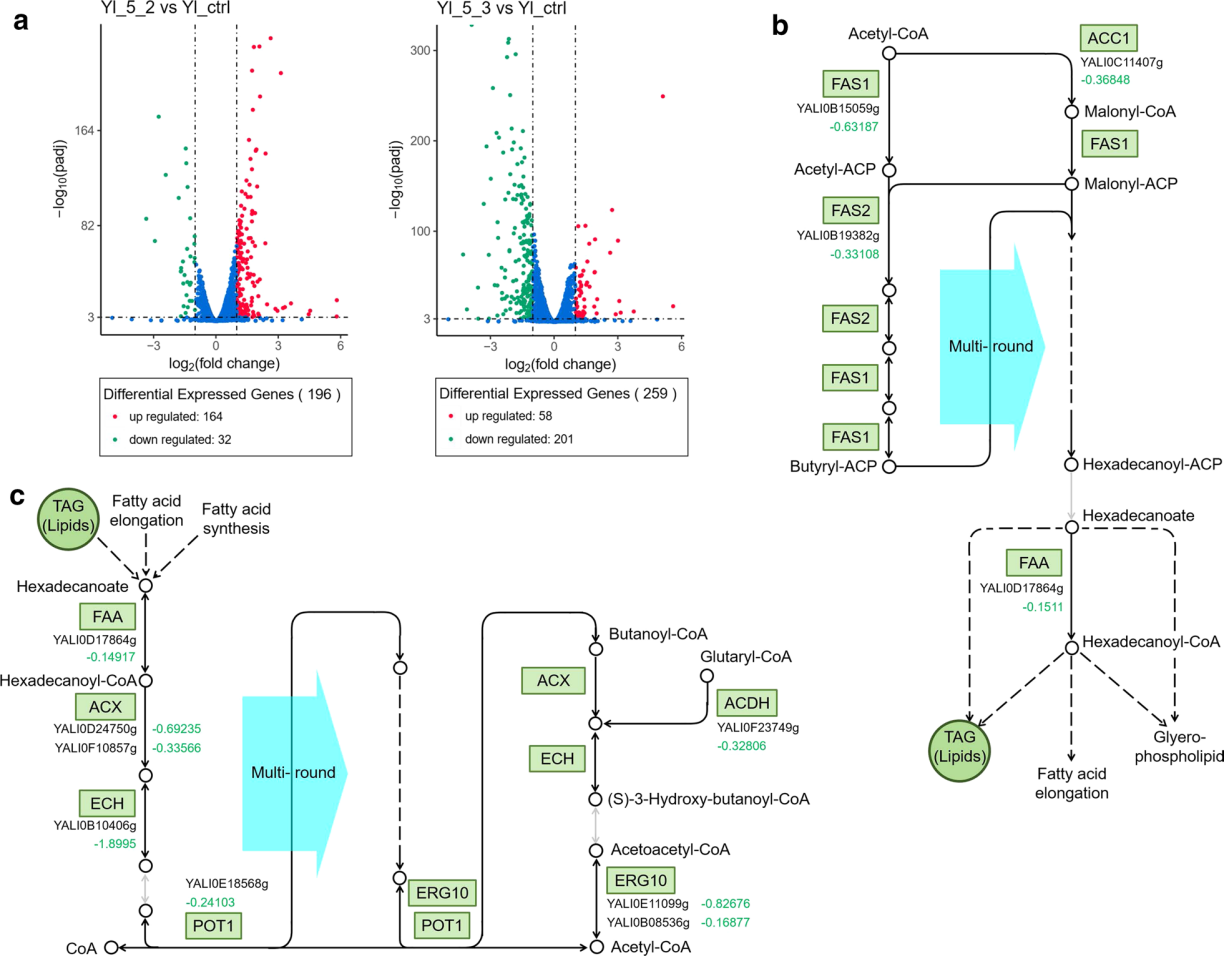
Comparative transcriptome analysis. **a** Most of the genes with differential expression in Yl_5_2 are up-regulated, while the majority in Yl_5_3 are down-regulated, indicating a whole “enhanced” transcription in Yl_5_2 but a whole “weakened” transcription in Yl_5_3. **b** The obviously down-regulated genes associated with fatty acid biosynthesis in Yl_5_2. **c** The obviously down-regulated genes associated with fatty acid degradation in Yl_5_3. The numbers under genes are log_2_(fold change of transcriptional read count). Three parallel samples for each strain were supplied for transcriptome analysis

In a recent work, the transcriptome analysis was done for *Y. lipolytica* strains producing heterologous lycopene or not [38]. Our work focused on the changes in different strains producing varied levels of beta-carotene. To reveal the effects of combinatorial mutation of *Yl*-*SPT15* on global transcription, the transcriptome analysis was done for the three strains of Yl_5_1 (as control), Yl_5_2, and Yl_5_3. Primarily, according to the Pearson Correlation analysis, the reasonableness of samples and reliability of the detection were confirmed (Additional file 1: Figure S8). The transcriptome data was analyzed under two conditions, a normal condition of *p* < 0.05, and a stringent condition of *p* < 0.001 and change-fold < 0.5 or > 2.0. According to the normal condition (*p* < 0.05) results, both Yl_5_2 and Yl_5_3 exhibited changed expressions of almost 3000 genes, nearly half of the total genes throughout genome (Additional file 1: Figure S9; Additional file 4). This ratio was coincident with the ratio of the genes under the control of Polymerase II transcriptional system in *S. cerevisiae*. This highly proved that *Yl*-*SPT15* did own the function of global TF. When the condition was stringent (*p* < 0.001 and fold < 0.5 or > 2.0), Yl_5_2 and Yl_5_3 exhibited altered expression of hundreds of genes throughout genome compared with the control (Fig. 3). Only one-tenth of these transcription-modulated genes (39 out of total 416) in two strains were in accordance, quite differentiated with that of normal condition (1943 out of total 4687). Most of the genes (164 out of total 196) with differential expression in Yl_5_2 were up-regulated, while the majority in Yl_5_3 (201 out of total 259) were down-regulated, indicating a whole “enhanced” transcription in Yl_5_2 but a whole “weakened” transcription in Yl_5_3. The highest two up-regulated targets in Yl_5_2 were *YALI0D17556g*(*RER2*) that expressed a major enzyme participating in polyprenol synthesis in both endoplasmic reticulum (ER) and lipid droplets, and *YALI0F19184g*(*RGT2*) that was plasma membrane high glucose sensor for regulating glucose transport.

The KEGG pathway enrichment was done for each pair of Yl_5_2 relative to Yl_5_1 and Yl_5_3 relative to Yl_5_1 (*p* < 0.05). The “enhanced” strain Yl_5_2 obtained some prominent enrichment of up-regulated genes in special pathways such as synthesis and degradation of ketone bodies, alpha-linolenic acid metabolism (an actual non-existing pathway in *Y. lipolytica* but related to three disperse real reactions later explained), mismatch repair, and homologous recombination (rich factors (RF) > 0.5) (Additional file 1: Figure S10 and Additional file 3: Table S4). The “weakened” strain Yl_5_3 gained some prominent enrichment of down-regulated genes in special pathways such as 10 amino acid metabolisms (valine, leucine, isoleucine, tryptophan, phenylalanine, tyrosine, lysine, histidine, cysteine, and methionine) and fatty acid degradation (RF > 0.6) (Additional file 1: Figure S11). The two strains shared an essential commonly changed pathway, RNA polymerase. The related genes in Y_5_2 were obviously down-regulated (RF = 0.62), while that in Yl_5_3 were notably up-regulated (RF = 0.55) (Additional file 1: Figures S12, S13). As mentioned above, another work concluded that the strain highly producing lipid bodies also highly produced carotene. However, in our work, a somewhat paradoxical phenomenon was observed. The genes associated with fatty acid biosynthesis in Yl_5_2 were obviously down-regulated (RF = 0.80), including the genes of *YALI0C11407g*(*ACC1*), *YALI0B15059g*(*FAS1*), *YALI0B19382g*(*FAS2*), and *YALI0D17864g*(*FAA*) (Additional file 1: Figure S13, Additional file 3: Table S5). By contrast, the fatty acid degradation was down-regulated in Yl_5_3 (RF = 0.78), including the genes of *YALI0D17864g*(*FAA*), *YALI0D24750*(*ACX*), *YALI0F10857g*(*ACX*), *YALI0B10406g*(*ECH*), *YALI0E18568g*(*POT1*), *YALI0E11099g*(*ERG10*), *YALI0B08536g*(*ERG10*), and *YALI0F23749g*(*ACDH*) (Additional file 1: Figure S11, Additional file 3: Table S6). There were two basic possibilities. First, the total carbon distribution to fatty acid synthesis and mevalonic acid (MVA) synthesis was constant and the increment of carotene caused down-regulated synthesis of fatty acids and vice versa. Second, the quantitative changes of carotene and fatty acids might be in accordance. The contradictory transcription regulation might indicate negative feedback regulation which commonly existed in fatty acid metabolism. The latter possibility was verified by later detection of the amounts of cellular fatty acids.

### Modulation of the amounts of total fatty acids, free fatty acids, and lipid bodies

**Fig. 4:**
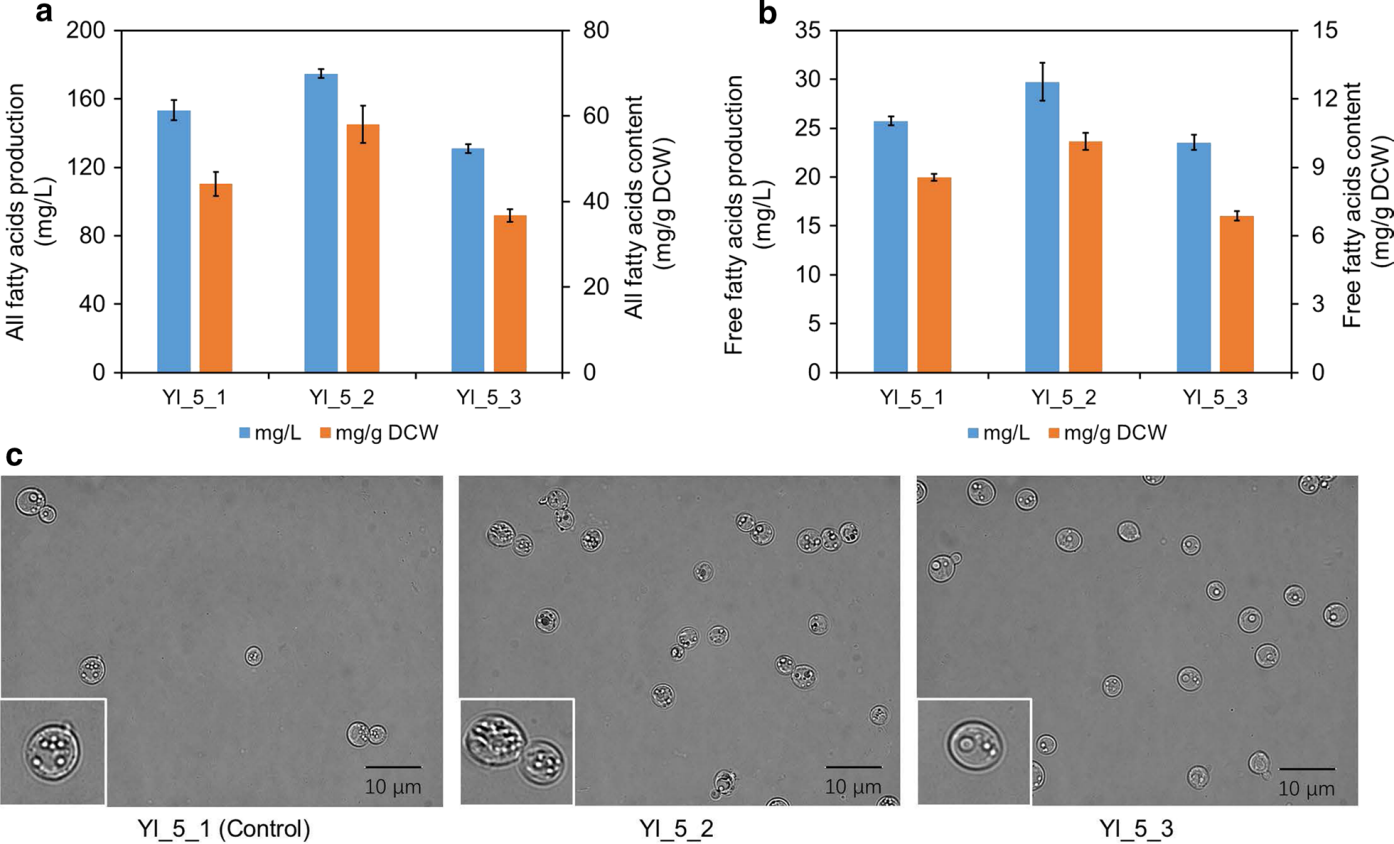
Detection of total fatty acids, free fatty acids, and lipid bodies. **a** The concentration of total fatty acids in Yl_5_2 was enhanced meanwhile that Yl_5_3 was reduced. **b** The free fatty acid concentrations in Yl_5_2 and Yl_5_3. **c** Lipid bodies in the strains were observed under microscopy, showing apparent variation. All error bars indicate ± standard deviation, *n* = 3

As transcriptome analysis revealed that fatty acid metabolism was regulated in both “enhanced” strain Yl_5_2 and “weakened” strain Yl_5_3, it was necessary to detect the amounts of fatty acids and lipids in these strains. The concentration of total fatty acids in Yl_5_2 was enhanced to 174.76 mg/L (58.03 mg/g DCW), 1.14-fold (1.32-fold) of the production contained in Yl_5_1 as 153.34 mg/L (44.11 mg/g DCW) (Fig. 4a, Additional file 1: Figure S14). As for free fatty acids, the concentration in Yl_5_2 was 29.73 mg/L (10.14 mg/g DCW), 1.15-fold (1.19-fold) of that in Yl_5_1 as 25.75 mg/L (8.55 mg/g DCW) (Fig. 4b, Additional file 1: Figure S15). By contrast, the concentration of total fatty acids in Yl_5_3 was reduced to 131.02 mg/L (36.73 mg/g DCW), 0.85-fold (0.83-fold) of that in Yl_5_1 (Fig. 4a, Additional file 1: Figure S14), and the free fatty acids was 23.54 mg/L (6.88 mg/g DCW) in Yl_5_3, 0.91-fold (0.80-fold) of that in Yl_5_1 (Fig. 4b, Additional file 1: Figure S15). The composition of FFA and total fatty acids was consistent among different strains (Additional file 1: Figures S14, S15). The increased production of both total and free fatty acids in Yl_5_2 might explain why its fatty acid synthesis pathway was down-regulated because this pathway owned typical mode of drastic feedback regulation by end products. On the other hand, the decreased production of fatty acids in Yl_5_3 explained why the fatty acid degradation pathway was down-regulated as cells had to down-regulate fatty acid degradation to protect its inner lipophilic environment. The microscopic observation showed that Yl_5_2 got more droplets of lipids but not a larger one compared with Yl_5_1 and Yl_5_3, as the increased lipophilic compartments were beneficial to carotene preservation and was created by whole cell’s complex tuning forces (Fig. 4c and Additional file 1: Figure S12).

**Fig. 5:**
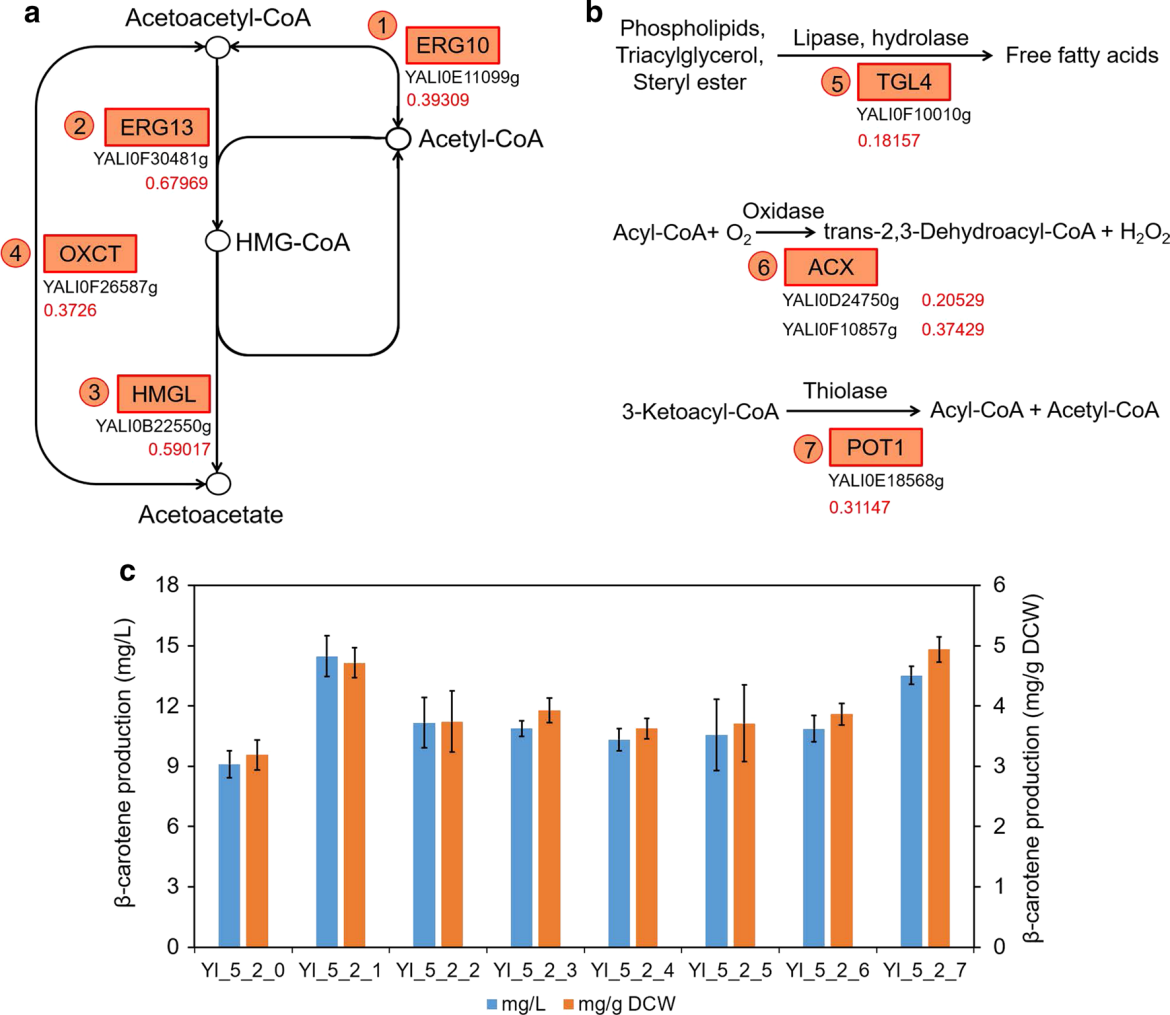
Effects of single native gene overexpression. **a** The genes associated with up-regulated pathway of ketone body metabolism in Yl_5_2. The numbers under genes are log_2_(fold change of transcriptional read count). **b** The genes associated with up-regulated alpha-linolenic acid metabolism in Yl_5_2. However, it did not exist in *Y. lipolytica*. The actual functions of the coupling of *ACX* and *POT1* shall be to produce final acetyl-CoA. Another gene *TGL4* charged degradation of multiple compounds to recycle free fatty acids. The numbers under genes are log_2_(fold change of transcriptional read count). **c** Seven typical genes were chosen from the up-regulated pathways in Yl_5_2 (relative to Yl_5_1) and transformed as one more cassette into the same strain. The results showed that all the seven transformed strains with recombinant plasmids gained higher beta-carotene production than the initial strain Yl_5_2 with blank plasmid (Yl_5_2_0). All error bars indicate ± standard deviation, *n* = 3

Among the transcriptional regulated genes, *ACC1*, *FAS1*, and *FAS2* played essential roles in the biosynthesis of fatty acids. The carboxylation of acetyl-CoA by *ACC1* was considered as rate-limiting step, and this gene was usually overexpressed for lipid accumulation in *S. cerevisiae* and *Y. lipolytica* [21, 24, 25, 39, 40]. Besides, the coordinate expression of *FAS1* and *FAS2* was necessary for heteromultimeric fatty acid synthase complex [41]. In *Y. lipolytica*, acyl-CoA was a key intermediate closely correlated with both free fatty acids and lipids (triacylglycerol, TAG), and its changing tendency was probably same as FFA and lipids (Fig. 4, Additional file 1: Figures S14, S15). As increased acyl-CoA plays the key role of negative feedback regulation of *ACC1*, it was understandable that the transcriptions of *YALI0C11407g*(*ACC1*), *YALI0B15059g*(*FAS1*), and *YALI0B19382g*(*FAS2*) were down-regulated in Yl_5_2 at the cell’s late growth stage. In addition, *YALI0E11099g*(*ERG10*) and *YALI0F10857g*(*ACX*) might play key roles as these two genes were regulated by obviously reverse trends in Yl_5_2 and Yl_5_3 (Figs. 4c, 5a, b). The two enzymes, acetyl-CoA C-acetyltransferase and acyl-CoA oxidase, also covered the crossroad locations for regulating distribution of acetyl-CoA flux in *Y. lipolytica*.

### Enhancement of carotene production by overexpressing newly revealed intrinsic genes

As previously mentioned, in this work we revealed new up-regulated pathways in Yl_5_2 that might be correlated with the phenotype of higher carotene production. To determine these obviously up-regulated pathways in Yl_5_2 contributed to the improved beta-carotene production, we chose seven typical up-regulated genes from the two pathways and transformed their individual expression cassette on plasmids into Yl_5_2 (Fig. 5a, b, Additional file 1: Figure S2c). The results showed that all the seven transformed strains with recombinant plasmids (from Yl_5_2_1 to Yl_5_2_7) gained higher beta-carotene production than the initial strain Yl_5_2 with blank plasmid (Yl_5_2_0). These genes did increase the flux of acetyl-CoA towards terpene synthesis. The increment was up to about 50–60%, although all the values were slightly reduced perhaps owing to the influences of cell’s growth on the medium with hygromycin B (Fig. 5c). The collaboration of four enzymes expressed individually from *YALI0E11099g*(*EGR10*), *YALI0F30481g*(*ERG13*), *YALI0B22550g*(*HMGL*), and *YALI0F26587g*(*OXCT*) generated a special recycling of acetyl-CoA in *Y. lipolytica* and its flux was obviously up-regulated in Yl_5_2 for enhancing the pull of acetyl-CoA towards HMG-CoA and offering more precursors to MVA pathway (Additional file 3: Table S4). The single-gene overexpression proved that the flux of this cycling could be further enhanced for improving carotene synthesis and therein the *YALI0E11099g*(*EGR10*) might be the bottleneck. Another up-regulated pathway alpha-linolenic acid metabolism was actually a mistake of KEGG enrichment analysis as it did not exist in *Y. lipolytica*. The actual functions of the coupling of *YALI0F10857g*(*ACX*) and *YALI0E18568*(*POT1*) shall be reacting repeated beta-oxidation of acyl-CoA to produce final acetyl-CoA (Fig. 5b, Additional file 3: Table S4). Another gene *YALI0F10010g*(*TGL4*) actually charged degradation of multiple compounds to recycle free fatty acids (Fig. 5b, Additional file 3: Table S4). Single-gene especially *YALI0E18568*(*POT1*) overexpression showed that it was beneficial to further enhance this pathway to gain more carotene products.

## Discussion

We have designed a strategy of global transcription engineering, like gTME, in *Y. lipolytica* for tuning lipophilic properties. The gTME promotes identification of genes requiring perturbation for optimizing cell’s complex phenotypes, which has been proved previously [6, 12, 34, 42]. Even recently, the heterologous transcriptional sigma factor has been modified to make *E. coli* accept amazing direct transcription of promoters from other prokaryotic resources. Here we constructed the coexistence of different ratios of wild-type and mutated *Yl*-*SPT15* genes in *Y. lipolytica*, permitting exploration of dominant mutations in the condition that other chromosomal genes could be unaltered. Fortunately, we obtained the assured changes of both genotype and phenotype in the selected strains with enhanced carotene production or weakened carotene production.

The results of transcriptional profiling revealed that the “enhanced” strain Yl_5_2 and the “weakened” strain Yl_5_3 exhibited differential expression of large amounts of genes relative to Yl_5_1 as control in the SC medium. Except for the P25T in Yl_5_2-module 5, the other four mutants all localized in the region from 194 to 216, which was corresponding to the region from 207 to 229 in *S. cerevisiae* (Additional file 1: Figure S1). Three out of total four mutants in Yl_5_2 (E208A in module 1 and A213T, A216V in module 5) were in “helix 2’” region. The only mutant F194L in Yl_5_3-module 2 was in the “repeat element 2” region. This phenomenon was coincident with previous work as this region was charged in the correlation with DNA sequence and other functional proteins [6, 35, 36].

The transcriptional changes in Yl_5_2 and Yl_5_3 were quite broad, exhibiting respective enrichment of certain pathways and functional gene groups (Fig. 3 and Additional file 1: Figures S10–S13). Yl_5_2 gained prominent enrichment of up-regulated pathways especially the metabolism of ketone bodies and three disperse reactions enriched in the fictitious alpha-linolenic pathway, but also a down-regulated pathway of fatty acid synthesis relative to Yl_5_1 (control). Yl_5_3 got prominent enrichment of down-regulated pathways especially up to 10 amino acid metabolisms and fatty acid degradation relative to Yl_5_1 (control). RNA polymerase was quite differentiated between the two strains as was down-regulated in Yl_5_2 but up-regulated in Yl_5_3. Further detection of total and free fatty acid contents in each selected strain proved that the total lipophilic products were tuned along with the tuned global transcription.

Seven genes in the up-regulated pathways with highest rich factors in Yl_5_2 were investigated as overexpression targets in this strain again. It was certified that the pathway of ketone body metabolism and three other disperse reactions were contributable to the improvement of beta-carotene production (Fig. 5). The results here showed difference from the *S. cerevisiae* gTME for screening strains with high production and tolerance of ethanol. In Alper’s work, no particular pathways or genetic networks were discovered as predominately responsible targets [6]. A reasonable explanation was that no stressed conditions were used in our work, although somewhat weakening the selection strength, providing chances to reveal those predominately and progressively tunable pathways under normal condition [37]. Our work offered some new targets to tune cell’s lipophilic products, as well as the previous approaches of molecular construction [21, 29]. It was a fact that the yields of beta-carotene in this work were still far lower than those in Gao’s work and Larroude’s work [23, 29]. Here only SC medium with minimal nutrients was used to cultivate cells, resulting not in optimal carotene production but offering a chance to research modes of tuning cellular lipophilic products.

**Fig. 6:**
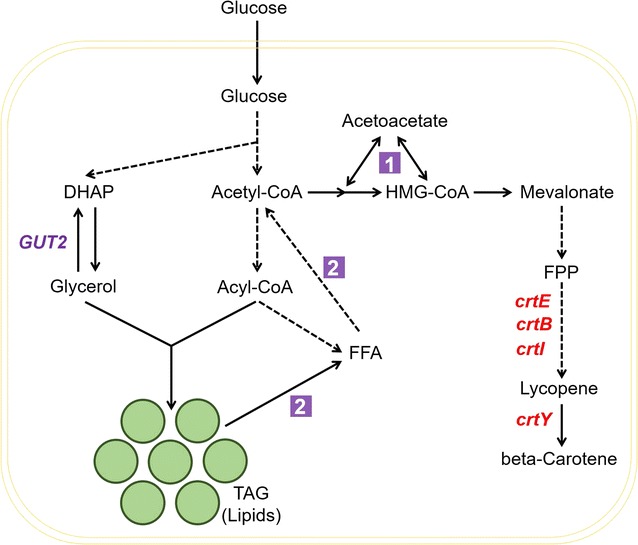
Model of tuning lipophilic properties in cells. A speculated model was built up for explaining the competitive relation between native lipid products and heterologous lipophilic product beta-carotene. The acetyl-CoA played a key role as a crossroad and was driven to beta-carotene synthesis in Yl_5_2 by the pathways 1 and 2 (in purple square). As a result, whole cell’s fatty acid products and lipophilic composition were tuned

A speculated model was that cells tuned special pathways to cope with competitive relations between lipophilic carotene and fatty products (Fig. 6). It was assured that there was a close correlation around the crossroad of acetyl-CoA between synthesis of carotenoids and fatty acid products, both as lipophilic properties sharing the common lipophilic space in cells. Primarily, the strain overproducing lipids also produced more beta-carotene after the complex tuning of global gene transcription. The proved highest effective resources of acetyl-CoA for synthesizing beta-carotene in Yl_5_2 were ketone body metabolism (pathway 1 in Fig. 6) and fatty acid beta-oxidation (pathway 2 in Fig. 6). This made the cells pull more fatty acids from lipid bodies. Meanwhile, the whole tuning tendency led to increment of lipid bodies generating more membrane structure versus volume, which might be beneficial to the storage of newly produced beta-carotene (Fig. 4c and Additional file 1: Figure S16). The driving force from pathway 1 and 2 also increased the amounts of free fatty acids and conversely down-regulated the synthesis pathway by negative feedback regulation [43]. The increment of fatty acids and the number of lipid droplets in the “enhanced” strain potentially indicated the phenotype of co-evolution of native and heterologous lipophilic product synthesis. Next, we would research on how the cell tunes itself between “multiple lipid droplets” stage and “single lipid droplet” stage. Our research offered another angle to study how to firstly construct a robust oil-rich chassis and then transfer it into a producer of target lipophilic products, as well as other strategies [30, 44].

## Conclusions

In this work a global transcription engineering strategy is designed and operated in *Y. lipolytica*, an oleaginous yeast. These results demonstrate the availability of gTME in this yeast for altering complex phenotypes such as lipophilicity. The synthesis of beta-carotene and fatty acid products can be meanwhile tuned by a close correlation model. The strategy is proved a promising tool for the gene manipulation in *Y. lipolytica* to optimize other desirable high value-added lipophilic metabolites.

## Methods

### Strains and media

The *Y. lipolytica* starting strain was ATCC 201249 (MATA ura3-302 leu2-270 lys8-11 PEX17-HA) from American Type Culture Collection (ATCC). All the strains and plasmids newly constructed in this work were given at Additional file 3: Table S7. *Y. lipolytica* cells were grown at 28 °C on petri dish (pH 6.5) or in liquid SC medium (pH 6.0) consisting of 20 g glucose, 6.7 g yeast nitrogen base (YNB), 2 g mixed amino acid powders only lacking both uracil and leucine (per liter). The fermentation was done as follows. First day, the cells were cultivated overnight. Next day, the cells were inoculated in new medium until 12 h and the harvested cells were inoculated again as initial OD = 0.1 in 50 mL fermentation medium in flask and cultivated for 96 h. *E. coli* competent cells (TransGen Biotech Company) for cloning DNA were grown at 37 °C in normal LB medium. Phosphatic buffer solution consisted of 137 mM NaCl, 2.7 mM KCl, 4.3 mM Na_2_HPO_4_, and 1.4 mM KH_2_PO_4_.

### Construction of *Yl*-*SPT15* mutant libraries

The mutation of *Yl*-*SPT15* was done by error-prone PCR. The wild-type *YL*-*SPT15* was primarily ligated in the plasmid pEASY-Blunt (TransGen Biotech Company). Then an error-prone PCR was operated using this constructed plasmid as template. The 100 µL PCR system contained 10 µL 10× FastTaq Buffer, 10 µL EP dNTP mixture (containing 5 mM dCTP, 5 mM dTTP, 2 mM dATG, and 2 mM dGTP), 0.05 mM M13F primer, 0.05 mM M13R primer, 2 µL FastTaq enzyme, 0.15 mM MnCl_2_, 1 µL template DNA, and ddH_2_O. The reaction was operated as follows: initial template denaturation at 94 °C for 3 min, followed by 30 cycles of denaturation at 94 °C for 30 s, primer annealing at 50 °C for 30 s, and elongation at 72 °C for 30 s, following that, a last amplification at 72 °C for 10 min was run. After that, the PCR products of *Yl*-*SPT15* mutant libraries were purified after gel electrophoresis and digested by *Bsm*BI enzyme. This digestion made all the DNA segments own standard left sticky end of 5′-AATG-3′ and right sticky end of 5′-TTTA-3′.

### Assembly of expression cassettes of *Yl*-*SPT15* libraries and integration into chromosome

After digested by *Bsm*BI, the mixed segments were ligated into five cassettes (Additional file 1: Figure S2) to get sequential expression modules. All the cassettes on plasmid pLD2 (modified from pEASY-Blunt with mutation of *Bsa*I and *Bsm*BI sites) owned standard left 5′-CATT-3′ and right 5′-TAAA-3′ sticky ends for ligation with *Yl*-*SPT15* libraries. The constructed expression cassettes were H3-GPATp-*Yl*-*SPT15* library-PEX16t-H4, H4-YAT1p-*Yl*-*SPT15* library-LIP1t-H5, H5-XPR2p-*Yl*-*SPT15* library-PEX20t-H6, H6-FBAp-*Yl*-*SPT15* library-CYC1t-H7, H7-LEUp-*Yl*-*SPT15* library-ACOt-H8. Most promoters and terminators were cloned according to Blazeck’s work except for some little modification [16]. All “Hx” were homologous arms at the length of 200 bp with low G/C contents (20–30%), which were all cloned directly from *S. cerevisiae* genome (primers were listed in Additional file 3: Table S2). The transformed *E. coli* colonies were scraped off the plates and plasmids were isolated. Then the cassettes were digested by *Not*I from plasmid backbones and the total DNA mixture was directly purified without running gel electrophoresis. 20 μL of each segment and an extra left module of “GUT2L-Leu2-H3” and a right module of “H8-GUT2R” were mixed to form a total 74 μL for further transformation into the *Y. lipolytica* strain already containing beta-carotene pathway (Additional file 1: Figure S2, the modules were constructed by a similar way and then assembled in yeast).

The “GUT2L” was 1000 bp upstream of *GUT2*’s ORF, and “GUT2R” was 1000 bp downstream of GUT2’s ORF. The *Y. lipolytica* transformation method was generally similar as the “LiAc/SS-DNA/PEG procedure” used for *S. cerevisiae* [45]. The total system was 360 μL including 74 μL DNA fragment, 10 μL boiled ss-carrier DNA (10 mg/mL), 240 μL 50% PEG, and 36 μl 1 M LiAc. The transformed cells were plated on SC–Ura–Leu plates and incubated for 3–4 days at 28 °C until the colonies were present.

### Detection of *Yl*-*SPT15* mutant genotypes in selected yeast strains

The yeast colonies with desired colors could be picked best after a week until the colors completely deposited. The genome of selected yeast cells was extracted using normal methods. The *Yl*-*SPT15* mutants in all five sequential cassettes were cloned from genome by a normal PCR method (Primers were shown in Additional file 3: Table S3). Also, the integration site GUT2 was verified using the upstream primer pair of “GUT2L” and “HR-F,” and downstream primer pair of “GUT2R” and “HR-R” (Additional file 3: Table S3). The PCR enzyme was FastPfu purchased from TransGen Biotech Company. The amplificated DNA products were purified after gel electrophoresis and sent to GENEWIZ Company for sequencing.

### Transcriptome analysis

The concentration of the extracted whole-cell RNA was measured with Qubit^®^ RNA Assay Kit in Qubit^®^ 2.0 Fluorometer (Life Technologies, CA, USA). Then an amount of 3 μg RNA per sample was used as input. Sequencing libraries were generated using NEBNext^®^ Ultra™ RNA Library Prep Kit for Illumina^®^ (NEB, USA). Finally, the PCR products were purified using AMPure XP system and the library quality was assessed by Agilent Bioanalyzer 2100 system. Then the libraries of PCR products were sequenced on an Illumina Hiseq 4000 platform and 150 bp paired-end reads were generated. The data analysis was all done in Novogene Company. Especially, the enrichment of differential expression genes in KEGG pathways (http://www.genome/jp/kegg/) was tested by KOBAS software.

### Extraction of cellular beta-carotene from *Y. lipolytica* cells

At the end of fermentation, the cells were collected and the supernatant was discarded after centrifugation. One milliliter of 3 M hydrochloric acid was added to the centrifuge tubes containing cells, shocked to blending. The centrifuge was put in boiling water for 5 min, then immediately put in ice bath for 5 min. The supernatant was discarded after centrifugation. Then the cells were washed with sterile water. One milliliter of acetone containing 1 g BHT (butylated hydroxytoluene) per liter was added to the cells and the tubes were shaken for 10 min. The supernatant was filtered with 0.2 μm hydrophobic membranes for collecting carotene products.

### Extraction of cellular total fatty acid from *Y. lipolytica* cells

Cells were harvested after centrifugation. One milliliter of methanol containing 3 M HCl and 10 mg/L heptadecanoic acid and 100 μL chloroform were added to the centrifuge tube containing cells, shocked to blending. Then the tubes were incubated at 70 °C for 3 h, reversed several times per 40 min. After that, the tubes were cooled down to room temperature. Then sodium chloride particle was added to take saturation, shaken for 1 min. After added with 500 μL hexyl hydride, the tube was shaken for a last minute. The supernatant was filtered with 0.2 μm hydrophobic membranes for collecting fatty ester products.

### Extraction of cellular free fatty acid from *Y. lipolytica* cells

800 μL of cell culture was taken into centrifuge tube from 96 h incubated cultures, then 400 μL dichloromethane containing 200 mM methyl iodide and 30 mg/L pentadecanoic acid as an internal standard was added into the tube and 20 μL 40% tetrabutylammonium hydroxide (base catalyst) was added immediately. The mixtures were shaken for 30 min at 1400–1600 rpm using a vortex mixer, and then centrifuged at 6000 rpm to promote phase separation. A 300 μL dichloromethane layer was transferred into a new centrifuge tube, and evaporated 1 h to dryness. The extracted methyl esters were resuspended in 150 μL hexane and then filtered with 0.2 μm hydrophobic membranes for collecting fatty ester products [46, 47].

### Measurement of beta-carotene

The quantitative determination of beta-carotene was achieved by HPLC. 10 μL sample was injected into a series e2695 HPLC, combined with a 2489 mass spectrometer (Waters, UV, USA), on a Ascentis^®^ Express C18 column (5 cm × 2.1 mm × 2.7 μm). The mobile phase consisted of 80% methanol, 18% acetonitrile, and 2% dichloromethane. HPLC conditions were mobile phase flow rate at 0.3 mL per min, sample room temperature programed at 22 °C, column temperature at 25 °C. The detection wavelength was at 450 nm. Every tested strain had three parallel samples. The beta-carotene standard sample was provided by SIGMA.

### Measurement of fatty acids

The quantitative detection of fatty acids was available by GC–MS method. 10 μL sample was injected into a series 6890N GC (Agilent Corp., USA), combined with a GCT Premier^™^ MICROMASS mass spectrometer (Waters Corp., USA), on a DB-5 ms column with a split injection of 5:1. GC conditions were carrier gas (helium) at 91 kPa per min in constant pressure mode, oven temperature programed from 70 °C (3 min) to 280 °C at the rate of 3 °C per min, source temperature at 250 °C, and interface temperature at 250 °C. Electron impact (EI) spectra were obtained at − 70 eV. GC–MS raw data were analyzed using the software package Masslynx4.1 (Waters Corp., USA), avoiding detector overload and isotope fractionation as described.

### Quantitative PCR

Q-PCR was carried out on a CFX96 Cycler-Real Time PCR Detection System (Bio-Rad Laboratories, Inc., Hercules, CA, USA), in white-walled PCR plates (96 wells). A ready-to-use master mix contained a fast proof-reading Polymerase, dNTPs, stabilizers, MgCl_2_, and SYBR^®^ Green dye and were used according to the manufacturer’s instructions (Bio-Rad). Reactions were prepared in a total volume of 18 μL containing 400 nM each primer (MWG), 2× SsoAdvanced™ SYBR^®^ Green Supermix (Bio-Rad), and 2 μL cDNA. The cycle conditions were set as follows: initial template denaturation at 95 °C for 3 min, followed by 40 cycles of denaturation at 95 °C for 10 s, and combined primer annealing/elongation at 57 °C for 20 s. The amount of fluorescence for each sample, given by the incorporation of SYBR^®^ Green into dsDNA, was measured at the end of each cycle and analyzed via CFX Manager™ software 2.1 (Bio-Rad Laboratories, Inc.). With Yl_ini as control, the copies of gene *GUT2* and *Yl*-*SPT15* were represented by the expression level of target gene divided by that of *ACT* gene which performed as the internal reference gene [48]. In another Q-PCR for quantification of carotene pathway gene integrations, the blank strain ATCC 201249 was chosen as control and the *ACT* was still used as a reference gene, in contrast, the Q-PCR was done on only genome extraction sample.

## Additional files

**Additional file 1: Figure S1.** Amino acid sequence alignment of *Yl-SPT15* (*YALI0B23056g*) in *Y. lipolytica* with *SPT15* in *S. cerevisiae*. The result shows their amino acid sequences are almost consistent. The red region is repeat element 1, the blue is helix 2, the yellow is repeat element 2, and the green is helix 2’. All these regions exhibite conserved characteristics between *Yl-SPT15* and *SPT15*. **Figure S2.** DNA manipulation toolkits. (a) The PCR products of *Yl-SPT15* mutant libraries were digested by *Bsm*BI enzyme and localized on the plasmid pLD-EcYl. (b) The manipulated *Yl-SPT15* mutant libraries were inserted in five expression cassettes and assembled meanwhile integrated in yeast chromosomal GUT2 site. (c) The *crtE*, *crtB*, *crtI* and *crtY* modules were assembled and integrated in chromosomal rDNA site (*crtE, crtB, crtI* from *Enterobacteriaceae bacterium*,* crtY* from *Pantoea ananas*). (d) Expression cassettes were constructed for the seven chosen endogenous genes and localized on the plasmid pLD-EcYl. pLD-EcYl was a newly constructed vector from pMCSCen1 (Blazeck, 2011) where a new hygromycin B resistance marker replaced previous URA marker. **Figure S3.** Correct assembly rate at GUT2 site. (a) A three-gene pathway (*crtE*, *crtB*, *crtI* from *Enterobacteriaceae bacterium*) was assembled and integrated in GUT2 site. (b) The numbers of correct red colonies and total colonies were counted after color verification by streaking on plates. (c) The correct assembly rates were calculated in the starting strain and in the strain with *ku70* knockout. All error bars indicate ±Standard Deviation, n = 3. **Figure S4.** The copy numbers of each cassette in strain Yl_ini were detected by Q-PCR. The gene *ACT* (YALI0D08272g) was chosen as an internal standard and the host strain ATCC 201249 as the negative control. The primers were designed to target promoters used in carotene pathway cassettes, namely EXP1p, TEFp, GPDp, GPATp. All the primers used for Q-PCR were listed in Table S21. All error bars indicate ±Standard Deviation, n = 3. **Figure S5.** The growth states and beta-carotene production of all strains. (a) The OD_600_ after cultivation for 96 h and production of beta-carotene of all the strains. Yl_1_1 owned one extra wild-type *Yl-SPT15* cassette at *GUT2* site based on Yl_ini. Similarly, Yl_3_1 owned three extra wild-type *Yl-SPT15* cassettes and Yl_4_1 owned four. (b)The growth curves of the strains under an unstressed condition with 20 g/L of glucose. All error bars indicate ±Standard Deviation, n = 3. **Figure S6.** Q-PCR detection of GUT2 site and *Yl-SPT15* in constructed strains. The expression of GUT2 was reduced to 0.643-fold (Yl_5_1), 0.501-fold (Yl_5_2) and 0.597-fold (Yl_5_3) detected by quantitative polymerase chain reaction (Q-PCR). While the expression of *Yl-SPT15* was similar, 0.895-fold (Yl_5_1), 0.886-fold (Yl_5_2) and 1.213-fold (Yl_5_3). As shown in the result, the expression of GUT2 was suppressed, while that of *Yl-SPT15* was almost conservative. The gene *ACT* (YALI0D08272g) was chosen as an internal standard and the strain Y_ini with *crtEBIY* pathway at rDNA site was used as control. All error bars indicate ±Standard Deviation, n = 3. **Figure S7.** An evaluation of the *Yl-SPT15* mutations in Yl_5_2 and Yl_5_3 on carotene production. (a) The exact single or combinatorial point mutants were introduced to natural *Yl-SPT15* gene by PCR and the expression cassette on plasmid pLD-EcYl was transformed into Yl_5_1. (b) The result illustrated that all mutations were contributive to the phenotype. The m0 meant the strain Yl_5_1 transformed with the plasmid containing wild-type *Yl-SPT15*. The m1b meant Yl_5_1 transformed with the plasmid containing mutation (Glu208Ala) localized in the first module of *Yl-SPT15* (as in Yl_5_2). The m5a meant Yl_5_1 transformed with the plasmid containing mutation (Pro25Thr) localized in the fifth module *Yl-SPT15* (as in Yl_5_2). The m5abc meant Yl_5_1 transformed with the plasmid containing mutations (Pro25Thr, Ala213Thr, Ala216Val) localized in the fifth module of *Yl-SPT15* (as in Yl_5_2). The m2 meant Yl_5_1 transformed with the plasmid containing mutation (Phe194Leu) localized in the second module of *Yl-SPT15* (as in Yl_5_3). **Figure S8.** Pearson correlation analysis of the control and the selected strains. The ctrl refers to Yl_5_1, the Y5_2 refers to Yl_5_2 and Y5_3 refers to Yl_5_3. Three parallel samples for each strain were supplied for transcriptome analysis. The nearer pearson correlation coefficients approximates to 1, the more similar its gene expression patterns are. Under ideal conditions, the square of Pearson’s correlation coefficient (R^2^) should be larger than 0.92, and our samples quite meet this requirement. All error bars indicate ±Standard Deviation, n = 3. **Figure S9.** Comparing with Yl_5_1, the transcriptions of almost half of the whole genome were regulated in Yl_5_2 and Yl_5_3 (p<0.05). **Figure S10.** The up-regulated pathways in Yl_5_2 relative to Yl_5_1 (Yl_ctrl). The synthesis and degradation of ketone bodies and alpha-linolenic acid metabolism are the most obviously up-regulated pathways in Yl_5_2. The up-regulated pathway alpha-linolenic acid metabolism was actually a mistake of KEGG enrichment analysis as it did not exist in *Y. lipolytica*. The actual functions of the coupling of *ACX*, *POT1* and *TGL4* was described in the article. **Figure S11.** The down-regulated pathways in Yl_5_3 relative to Yl_5_1 (Yl_ctrl). Fatty acid degradation and 10 amino acids metabolism are down-regulated distinctly in Yl_5_3 relative to Yl_5_1. **Figure S12.** The up-regulated pathways in Yl_5_3 relative to Yl_5_1 (Yl_ctrl). RNA polymerase pathway is obviously up-regulated in Yl_5_3 to Yl_5_1. **Figure S13.** The down-regulated pathways in Yl_5_2 relative to Yl_5_1 (Yl-ctrl). RNA polymerase and fatty acid biosynthesis are the most obviously down-regulated pathways in Yl_5_2 relative to Yl_5_1. **Figure S14.** The total contents of various fatty acids in each strain. There are six kinds of fatty acids that has been obviously detected in the strains and their contents are quite different between the selected strains. All error bars indicate ±Standard Deviation, n  = 3. **Figure S15.** The contents of various free fatty acids in each strain. There are five kinds of free fatty acids that has been obviously detected in the strains and their contents are quite different between the selected strains. All error bars indicate ±Standard Deviation, n = 3. **Figure S16.** Photomicrograph of lipid bodies after stained with sudan black B. Lipid bodies were observed under microscopy after stained with sudan black B and appeared dark. The photos showed that the Yl_5_2 strain contained more droplets of lipid bodies than Yl_5_1 as control and Yl_5_3. Cell staining method: The cells were collected after centrifugation and stained with sudan black B for 90 seconds, then the cells were washed with 70% ethanol for twice and suspended with sterile water.

**Additional file 2.** Supporting Online Texts.

**Additional file 3: Table S1.** Oligonucleotide primers used for Q-PCR. **Table S2.** Primers for PCR amplification to identify the genotypes of mutants. **Table S3.** Primers for PCR amplification to identify the genotypes of mutants and the insertion site. **Table S4.** Up-regulated genes in the pathways of ketone bodies and alpha-linolenic acid metabolism in Yl_5_2. **Table S5.** Down-regulated genes in the pathway of fatty acid biosynthesis in Yl_5_2. **Table S6.** Down-regulated genes in the pathway of fatty acid degradation in Yl_5_3. **Table S7.** All strains can be obtained according to their standard numbers in laboratory.

**Additional file 4.**Transcription heat map. The differential expressed genes are listed (*p* < 0.05).

## Abbreviations

*S. cerevisiae*: *Saccharomyces cerevisiae*
*Y. lipolytica*: *Yarrowia lipolytica*
gTME: global transcription machinery engineering
TF: transcription factor
TAG: triglyceride
PCR: polymerase chain reaction
HR: homologous recombination
RF: rich factor
