# Supplementary material for: Engineering global transcription to tune lipophilic properties in Yarrowia lipolytica
Source: Biotechnol Biofuels. 2018 Apr 19;11:115. doi: 10.1186/s13068-018-1114-z (PMC5907459; doi:10.1186/s13068-018-1114-z)
Supplement: Supplementary file 1 — Additional file 1: Figure S1. Amino acid sequence alignment of Yl-SPT15 (YALI0B23056g) in Y. lipolytica with SPT15 in S. cerevisiae. The result shows their amino acid sequences are almost consistent. The red region is repeat element 1, the blue is helix 2, the yellow is repeat element 2, and the green is helix 2’. All these regions exhibite conserved characteristics between Yl-SPT15 and SPT15. Figure S2. DNA manipulation toolkits. (a) The PCR products of Yl-SPT15 mutant libraries were digested by BsmBI enzyme and localized on the plasmid pLD-EcYl. (b) The manipulated Yl-SPT15 mutant libraries were inserted in five expression cassettes and assembled meanwhile integrated in yeast chromosomal GUT2 site. (c) The crtE, crtB, crtI and crtY modules were assembled and integrated in chromosomal rDNA site (crtE, crtB, crtI from Enterobacteriaceae bacterium, crtY from Pantoea ananas). (d) Expression cassettes were constructed for the seven chosen endogenous genes and localized on the plasmid pLD-EcYl. pLD-EcYl was a newly constructed vector from pMCSCen1 (Blazeck, 2011) where a new hygromycin B resistance marker replaced previous URA marker. Figure S3. Correct assembly rate at GUT2 site. (a) A three-gene pathway (crtE, crtB, crtI from Enterobacteriaceae bacterium) was assembled and integrated in GUT2 site. (b) The numbers of correct red colonies and total colonies were counted after color verification by streaking on plates. (c) The correct assembly rates were calculated in the starting strain and in the strain with ku70 knockout. All error bars indicate ±Standard Deviation, n = 3. Figure S4. The copy numbers of each cassette in strain Yl_ini were detected by Q-PCR. The gene ACT (YALI0D08272g) was chosen as an internal standard and the host strain ATCC 201249 as the negative control. The primers were designed to target promoters used in carotene pathway cassettes, namely EXP1p, TEFp, GPDp, GPATp. All the primers used for Q-PCR were listed in Table S21. All error bars indicate [file 13068_2018_1114_MOESM1_ESM.docx]

**Additional file 1: Figures S1-S16**


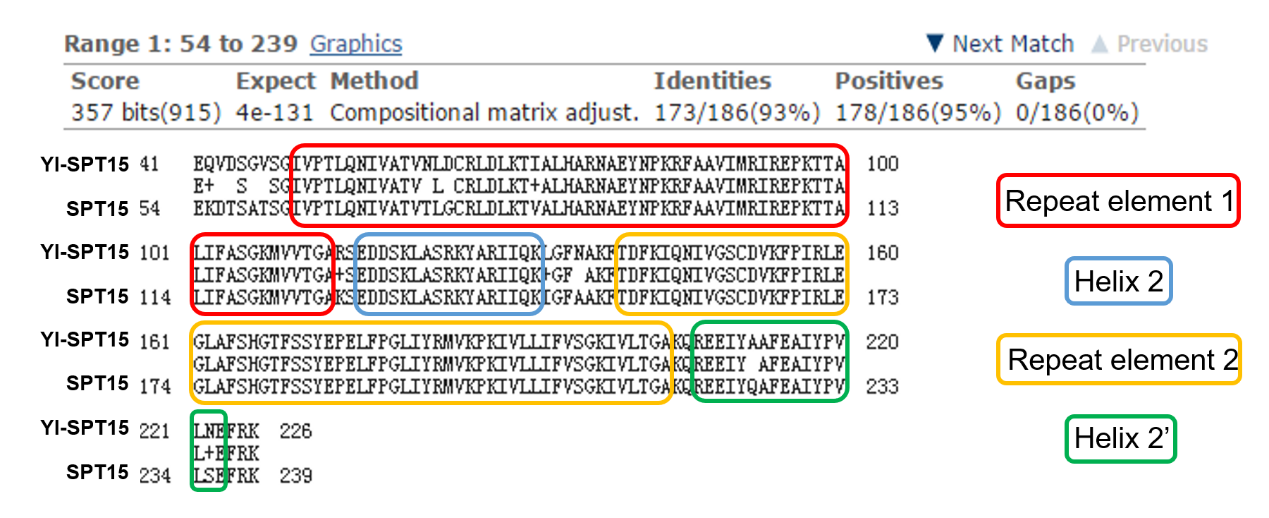


**Figure S1:** **Amino acid sequence alignment of *Yl-SPT15* (YALI0B23056g) in *Y. lipolytica* with *SPT15* in *S. cerevisiae*.** The result shows their amino acid sequences are almost consistent. The red region is repeat element 1, the blue is helix 2, the yellow is repeat element 2, and the green is helix 2’. All these regions exhibite conserved characteristics between *Yl-SPT15* and *SPT15*.


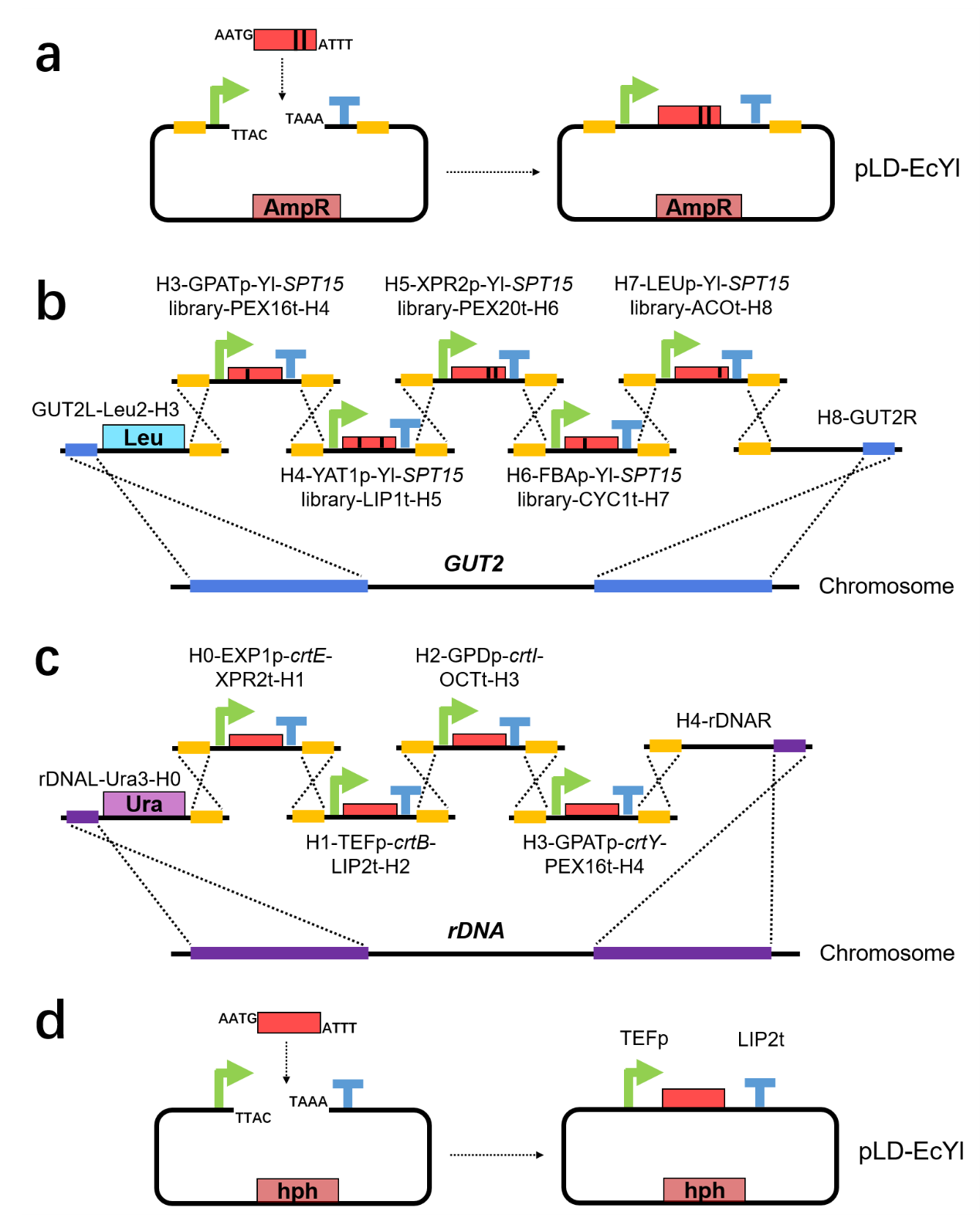


**Figure S2: DNA manipulation toolkits.** (a) The PCR products of *Yl-SPT15* mutant libraries were digested by *Bsm*BI enzyme and localized on the plasmid pLD-EcYl. (b) The manipulated *Yl-SPT15* mutant libraries were inserted in five expression cassettes and assembled meanwhile integrated in yeast chromosomal GUT2 site. (c) The *crtE*, *crtB*, *crtI* and *crtY* modules were assembled and integrated in chromosomal rDNA site (*crtE*, *crtB*, *crtI* from *Enterobacteriaceae bacterium*, *crtY* from *Pantoea  ananatis*). (d) Expression cassettes were constructed for the seven chosen endogenous genes and localized on the plasmid pLD-EcYl. pLD-EcYl was a newly constructed vector from pMCSCen1 (Ref.16 in text) where a new hygromycin B resistance marker replaced previous URA marker.


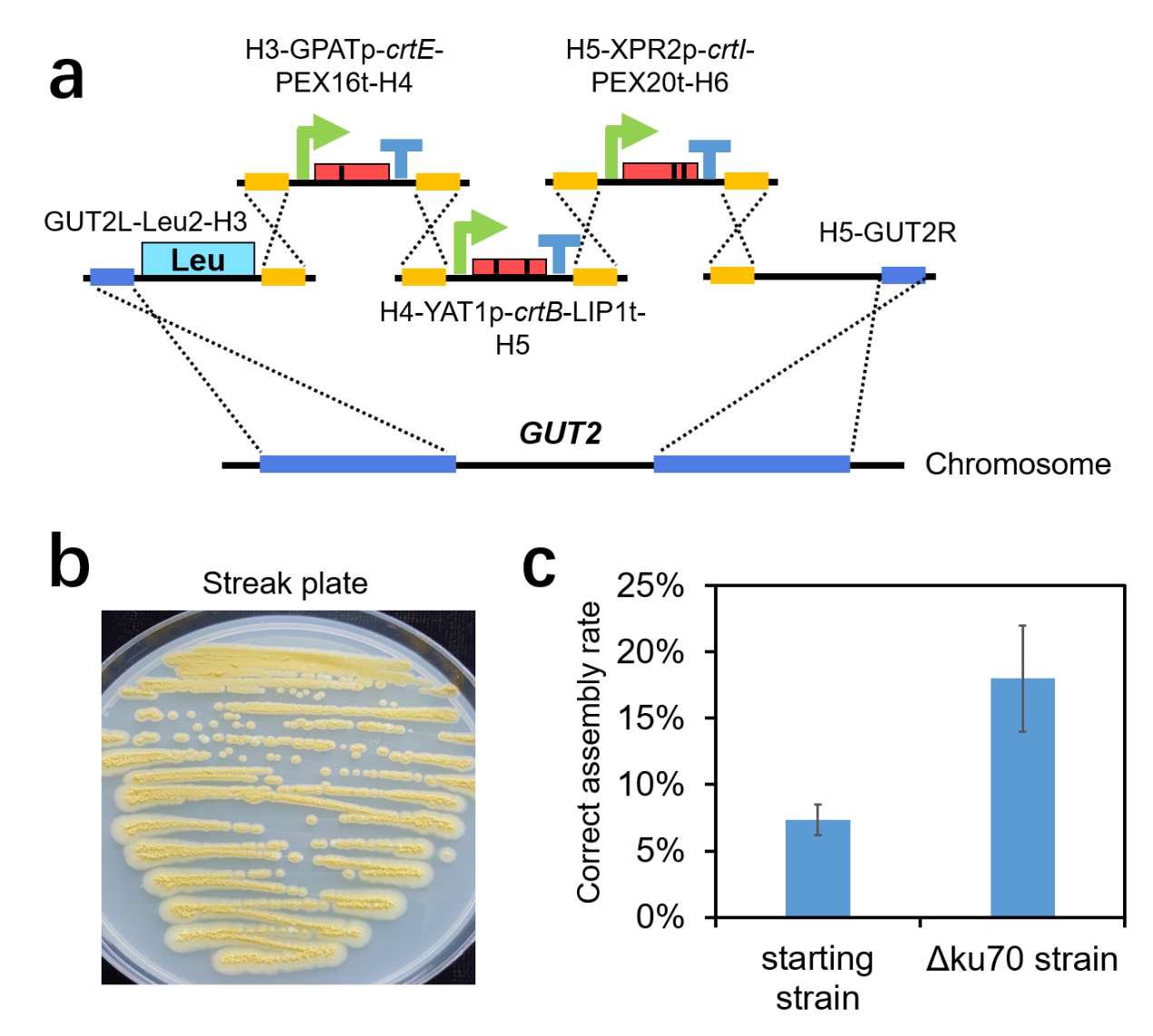


**Figure S3: Correct assembly rate at GUT2 site.** (a) A three-gene pathway (*crtE*, *crtB*, *crtI* from *Enterobacteriaceae bacterium*) was assembled and integrated in GUT2 site. (b) The numbers of correct red colonies and total colonies were counted after color verification by streaking on plates. (c) The correct assembly rates were calculated in the starting strain and in the strain with *ku70* knockout. All error bars indicate ±Standard Deviation, n=3.

**
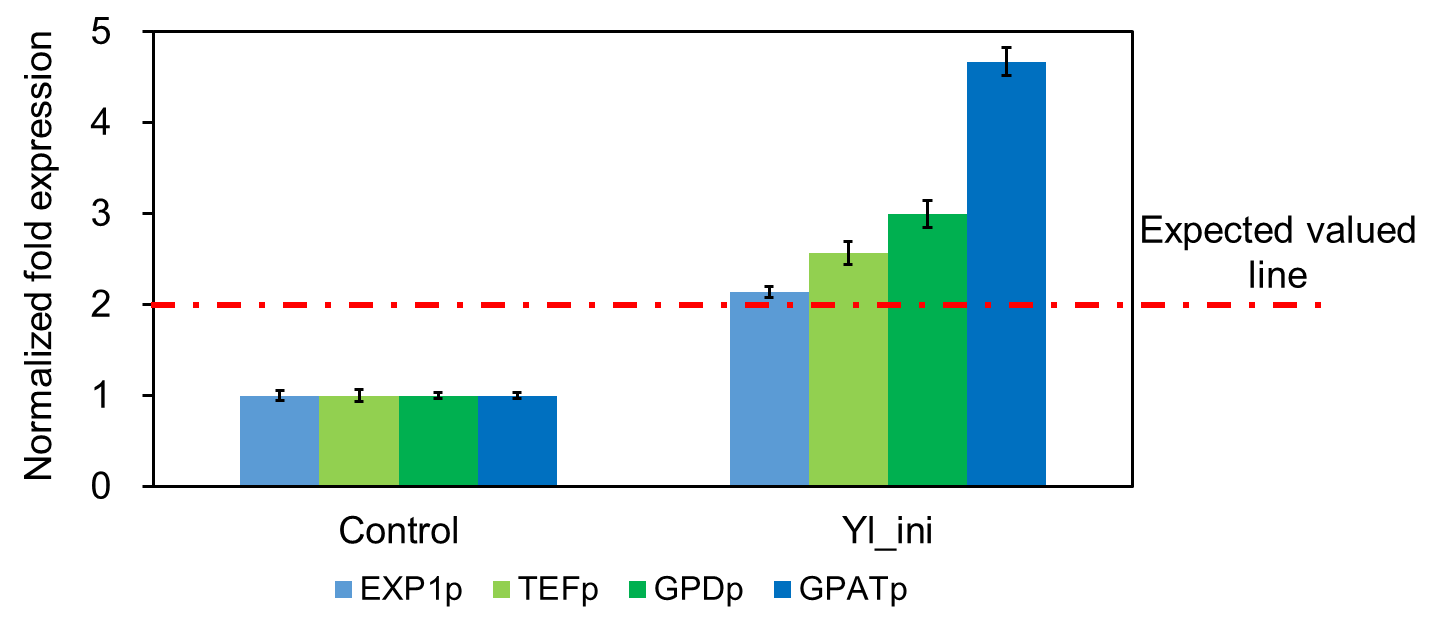
**

**Figure S4: The copy numbers of each cassette in strain Yl_ini were detected by Q-PCR .** The gene *ACT* (YALI0D08272g) was chosen as an internal standard and the host strain ATCC 201249 as the negative control. The primers were designed to target promoters used in carotene pathway cassettes, namely EXP1p, TEFp, GPDp, GPATp. All the primers used for Q-PCR were listed in Table S2. All error bars indicate ±Standard Deviation, n=3.


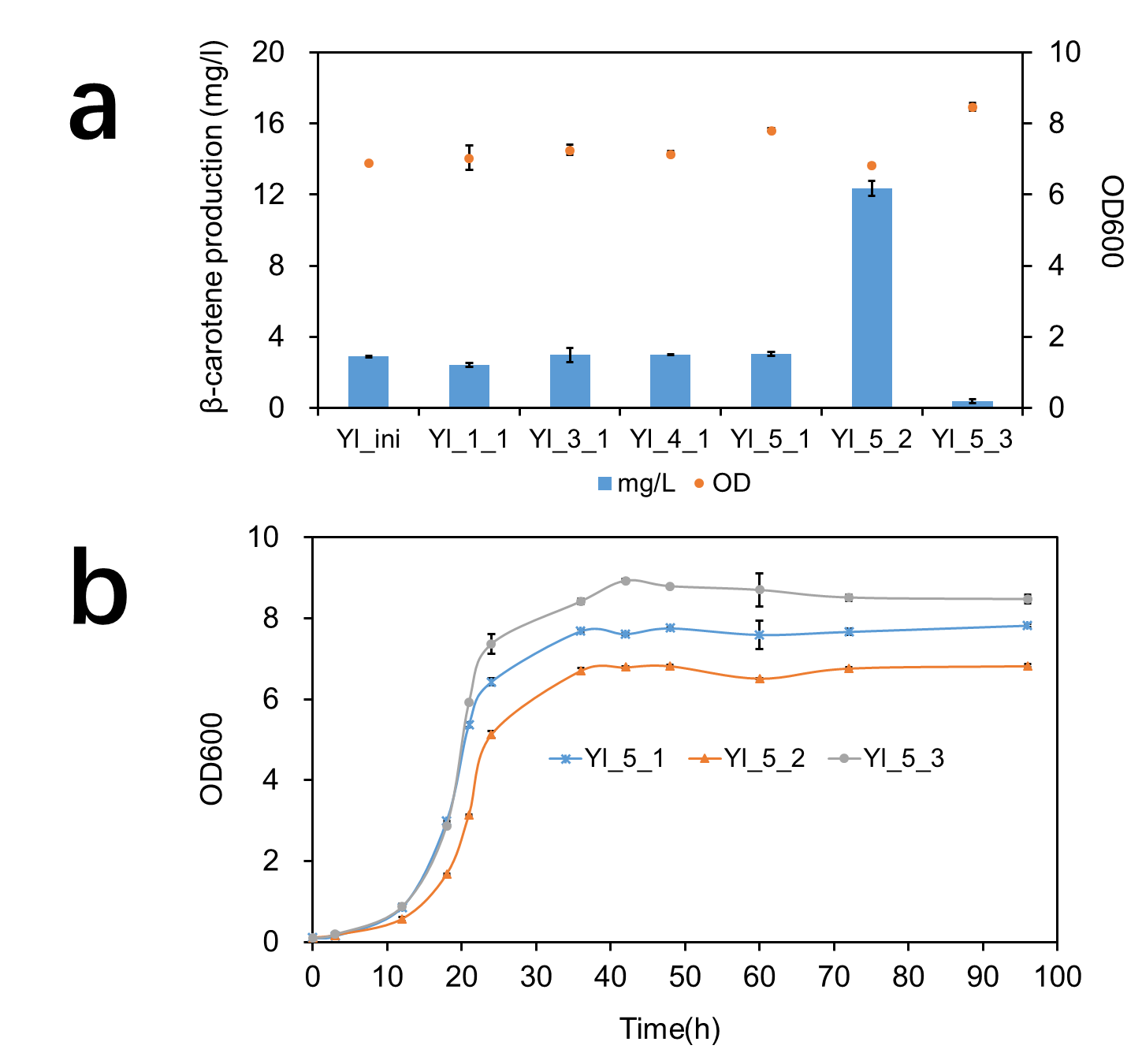


**Figure S5: The growth states and** **beta-carotene production of all strains.** (a) The OD_600_ after cultivation for 96 h and production of beta-carotene of all the strains. Yl_1_1 owned one extra wild-type *Yl-SPT15* cassette at *GUT2* site based on Yl_ini. Similarly, Yl_3_1 owned three extra wild-type *Yl-SPT15* cassettes and Yl_4_1 owned four. (b)The growth curves of the strains under an unstressed condition with 20 g/L of glucose. All error bars indicate ±Standard Deviation, n=3.

**
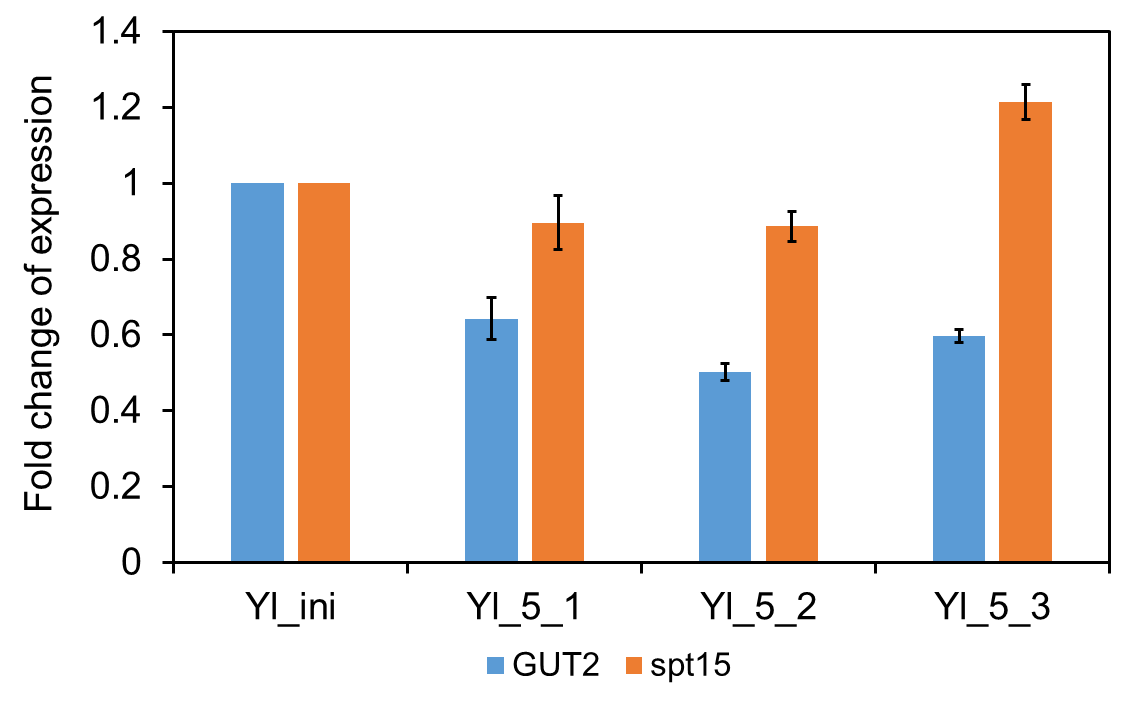
**

**Figure S6:** **Q-PCR detection of GUT2 site and *Yl-SPT15* in constructed strains.** The expression of GUT2 was reduced to 0.643-fold (Yl_5_1), 0.501-fold (Yl_5_2) and 0.597-fold (Yl_5_3) detected by quantitative polymerase chain reaction (Q-PCR). While the expression of *Yl-SPT15* was similar, 0.895-fold (Yl_5_1), 0.886-fold (Yl_5_2) and 1.213-fold (Yl_5_3). As shown in the result, the expression of GUT2 was suppressed, while that of *Yl-SPT15* was almost conservative. The gene *ACT* (YALI0D08272g) was chosen as an internal standard and the strain Y_ini with *crtEBIY* pathway at rDNA site was used as control. All error bars indicate ±Standard Deviation, n=3.

**
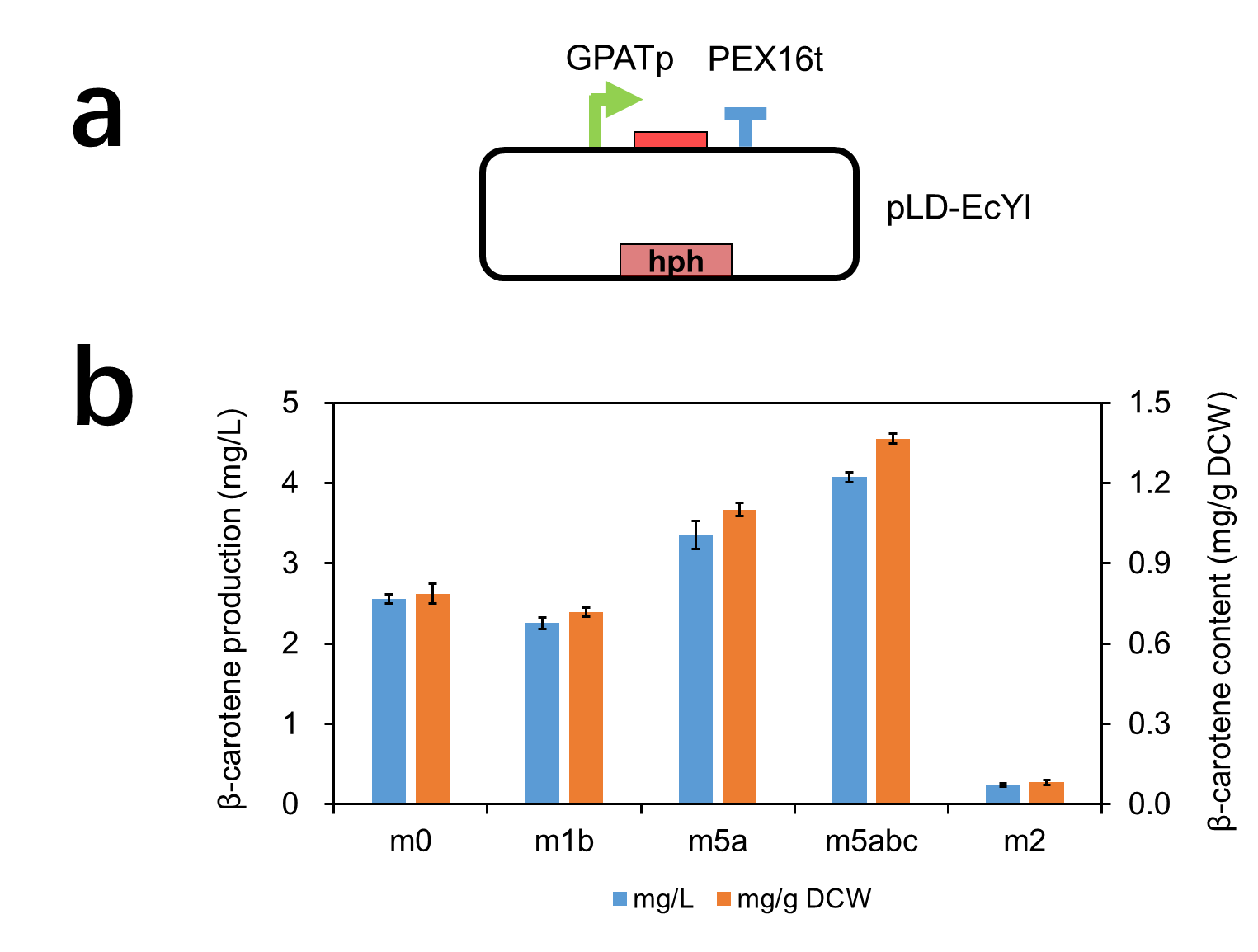
**

**Figure S7:** **An evaluation of the *Yl-SPT15* mutations in Yl_5_2 and Yl_5_3 on carotene production.** (a) The exact single or combinatorial point mutants were introduced to natural *Yl-SPT15* gene by PCR and the expression cassette on plasmid pLD-EcYl was transformed into Yl_5_1. (b) The result illustrated that all mutations were contributive to the phenotype. The m0 meant the strain Yl_5_1 transformed with the plasmid containing wild-type *Yl-SPT15*. The m1b meant Yl_5_1 transformed with the plasmid containing mutation (Glu208Ala) localized in the first module of *Yl-SPT15* (as in Yl_5_2). The m5a meant Yl_5_1 transformed with the plasmid containing mutation (Pro25Thr) localized in the fifth module *Yl-SPT15* (as in Yl_5_2). The m5abc meant Yl_5_1 transformed with the plasmid containing mutations (Pro25Thr, Ala213Thr, Ala216Val) localized in the fifth module of *Yl-SPT15* (as in Yl_5_2). The m2 meant Yl_5_1 transformed with the plasmid containing mutation (Phe194Leu) localized in the second module of *Yl-SPT15* (as in Yl_5_3).


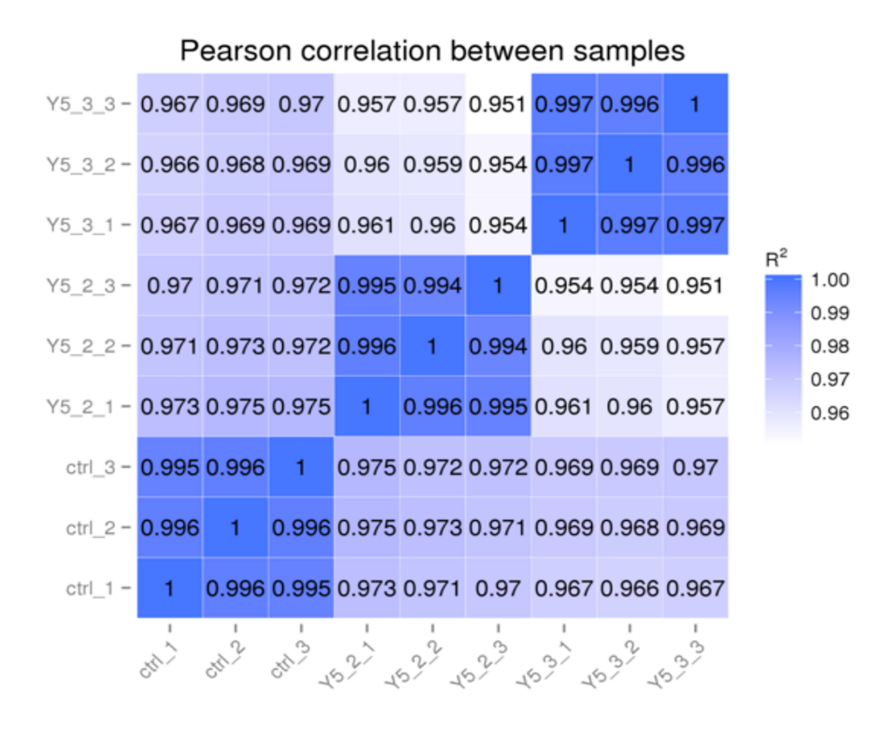


**Figure S8:** **Pearson correlation analysis of the control and the selected strains.** The ctrl refers to Yl_5_1, the Y5_2 refers to Yl_5_2 and Y5_3 refers to Yl_5_3. Three parallel samples for each strain were supplied for transcriptome analysis. The nearer pearson correlation coefficients approximates to 1, the more similar its gene expression patterns are. Under ideal conditions, the square of Pearson's correlation coefficient (R^2^) should be larger than 0.92, and our samples quite meet this requirement.

**
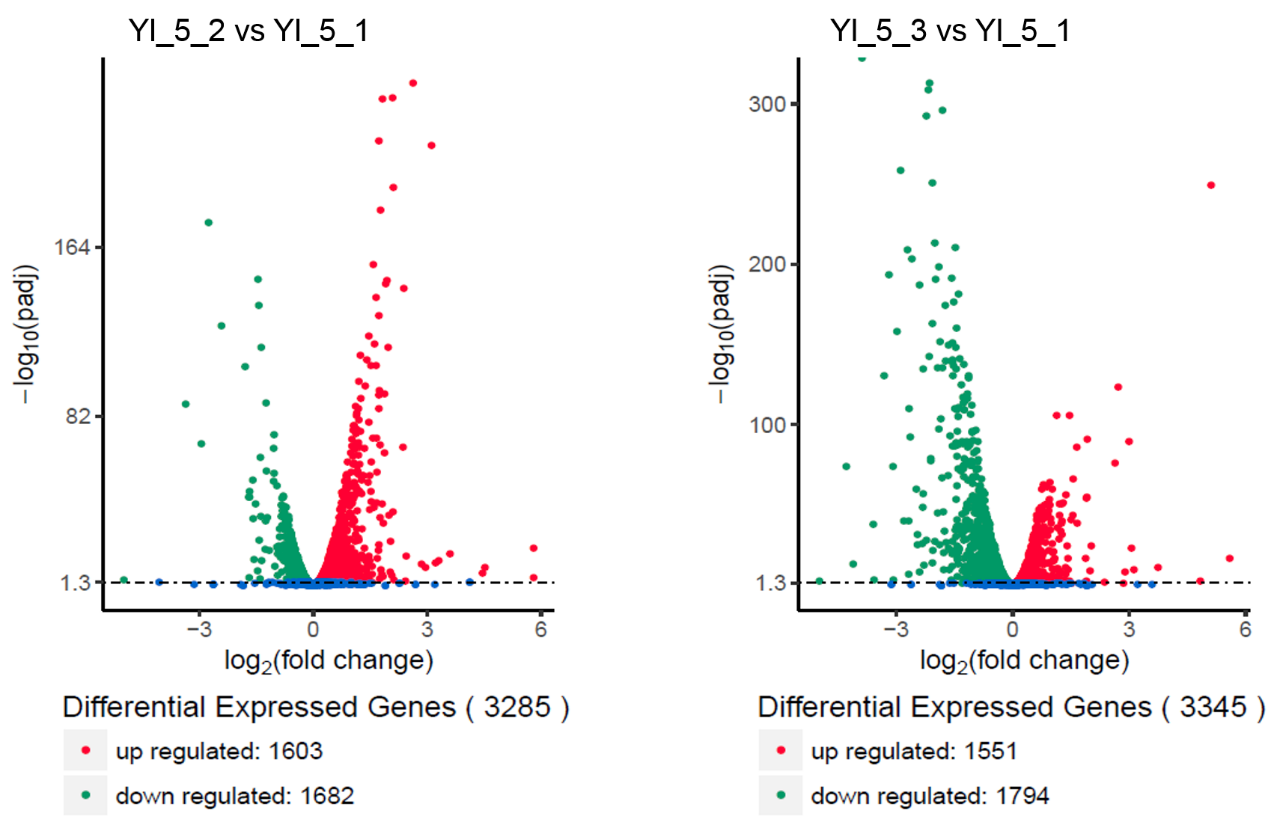
**

**Figure S9:** Comparing with Yl_5_1, the transcriptions of almost half of the whole genome were regulated in Yl_5_2 and Yl_5_3 (p<0.05).

**
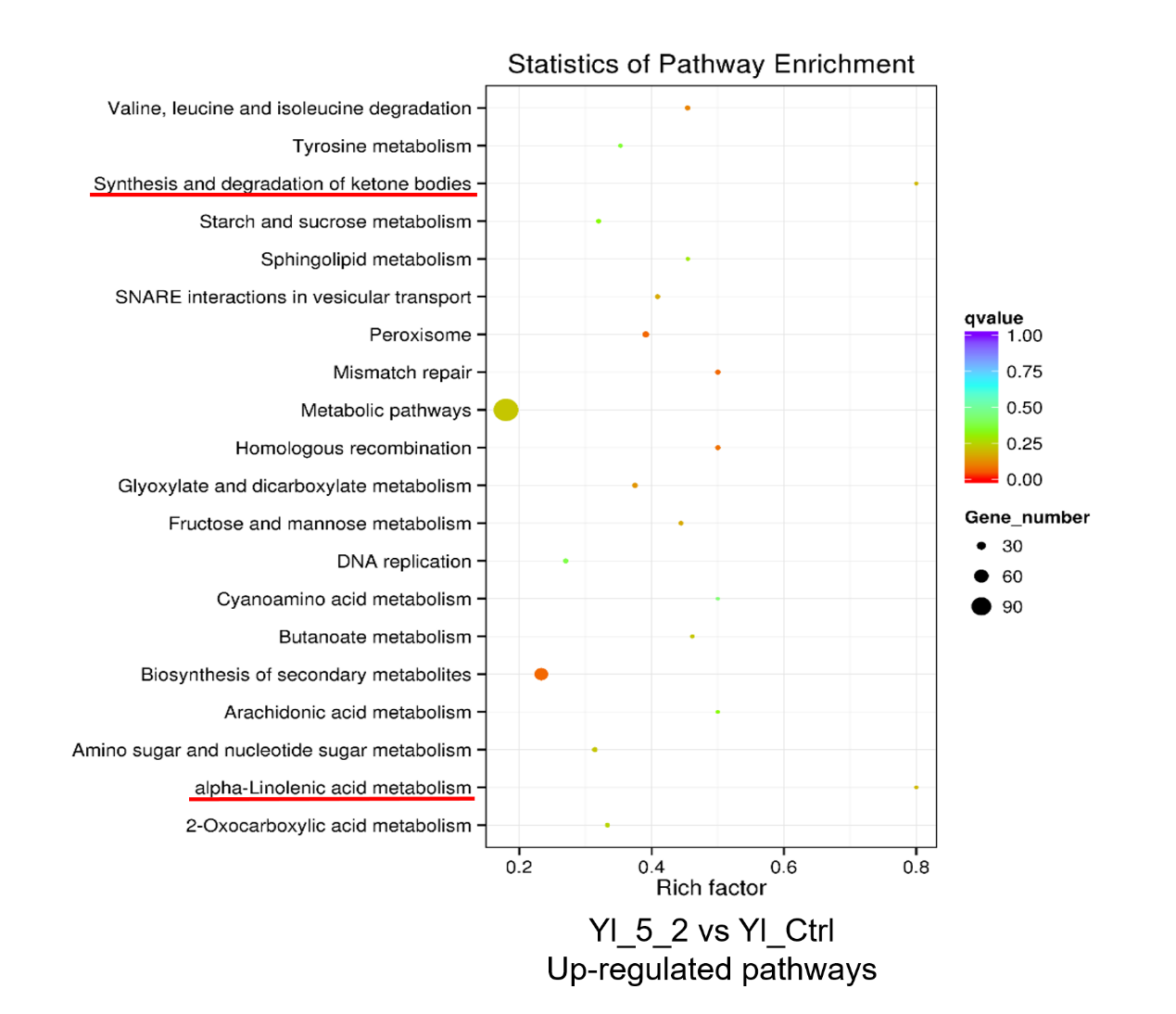
**

**Figure S10:** **The up-regulated pathways in Yl_5_2 relative to Yl_5_1 (Yl_ctrl).** The synthesis and degradation of ketone bodies and alpha-linolenic acid metabolism are the most obviously up-regulated pathways in Yl_5_2 relative to Yl_5_1. The up-regulated pathway alpha-linolenic acid metabolism was actually a mistake of KEGG enrichment analysis as it did not exist in *Y. lipolytica*. The actual functions of the coupling of *ACX*, *POT1* and *TGL4* were described in the article.

**
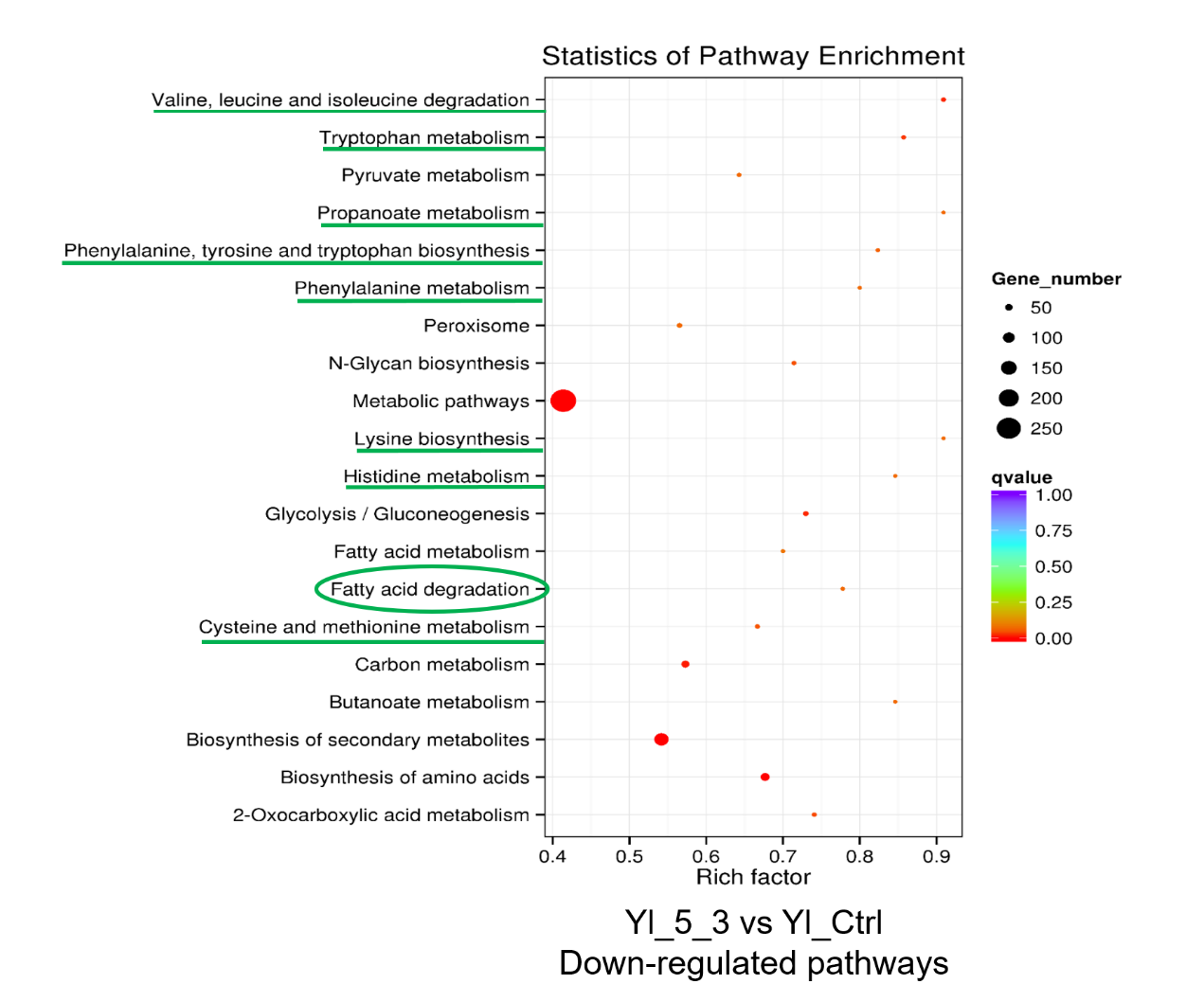
**

**Figure S11: The down-regulated pathways in Yl_5_3 relative to Yl_5_1 (Yl_ctrl).** Fatty acid degradation and 10 amino acids metabolism are down-regulated distinctly in Yl_5_3 relative to Yl_5_1.

**
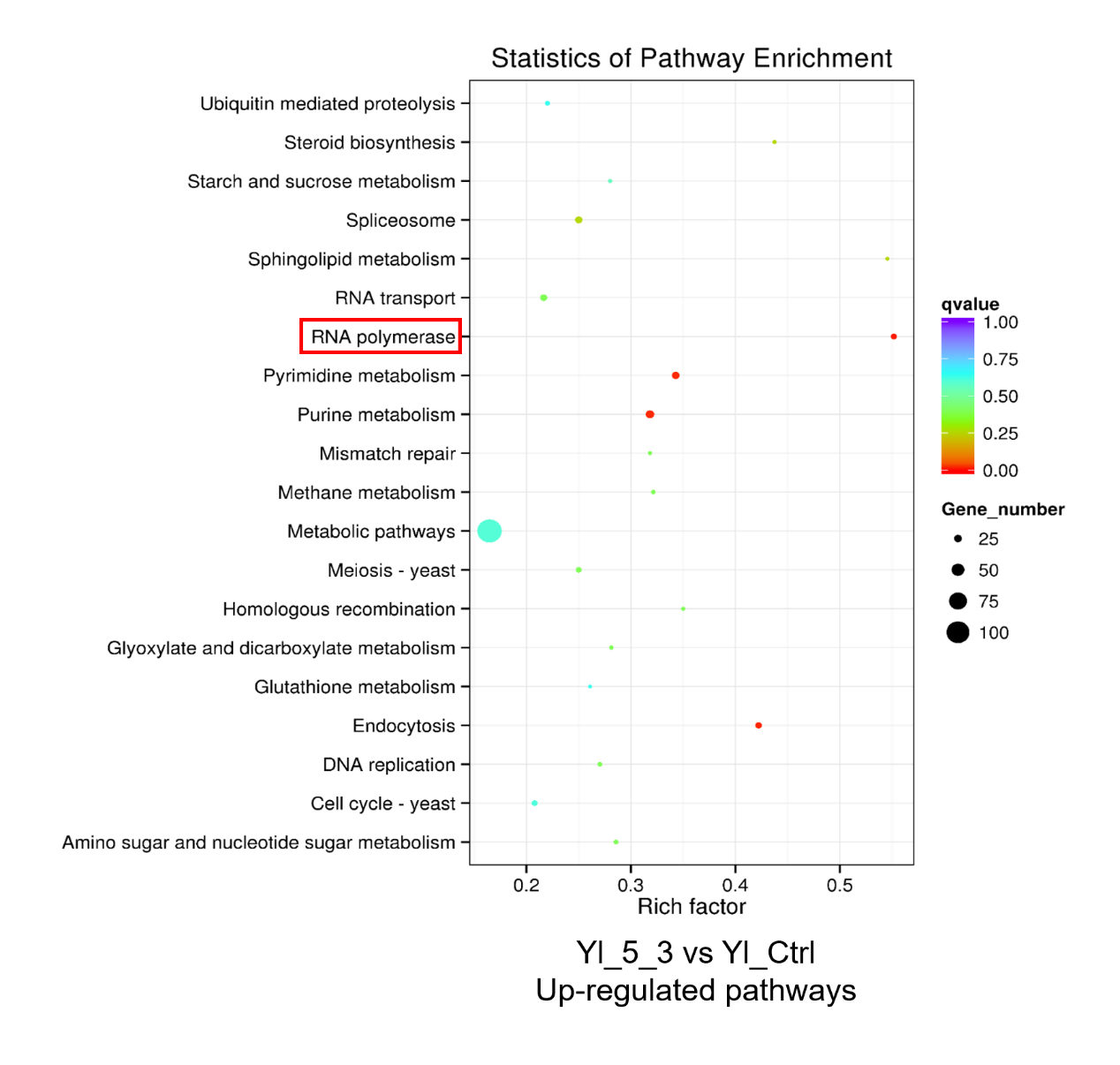
**

**Figure S12:** **The up-regulated pathways in Yl_5_3 relative to Yl_5_1 (Yl_ctrl).** RNA polymerase pathway is obviously up-regulated in Yl_5_3 to Yl_5_1.

**
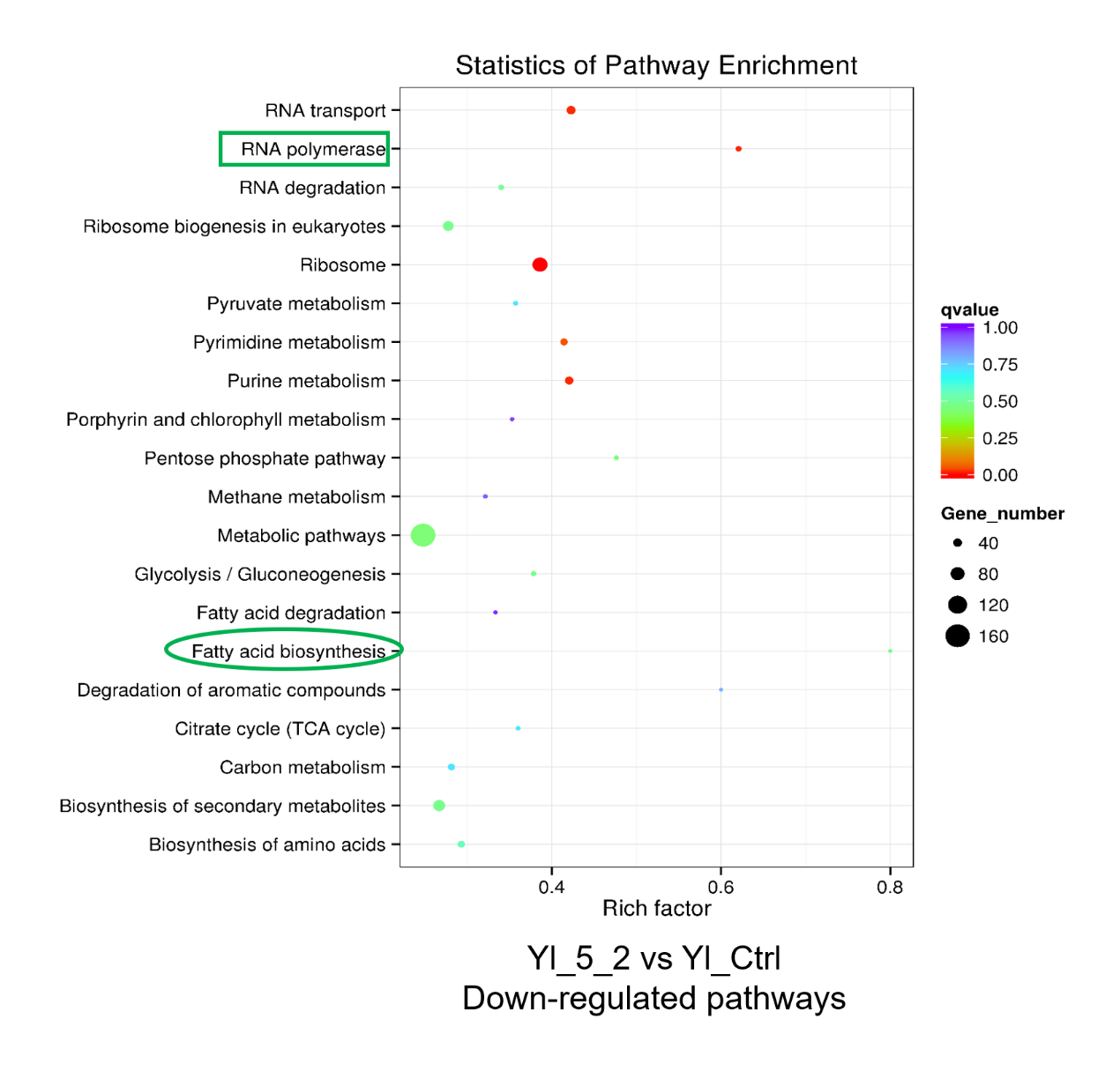
**

**Figure S13: The down-regulated pathways in Yl_5_2 relative to Yl_5_1 (Yl-ctrl).** RNA polymerase and fatty acid biosynthesis are the most obviously down-regulated pathways in Yl_5_2 relative to Yl_5_1.

**
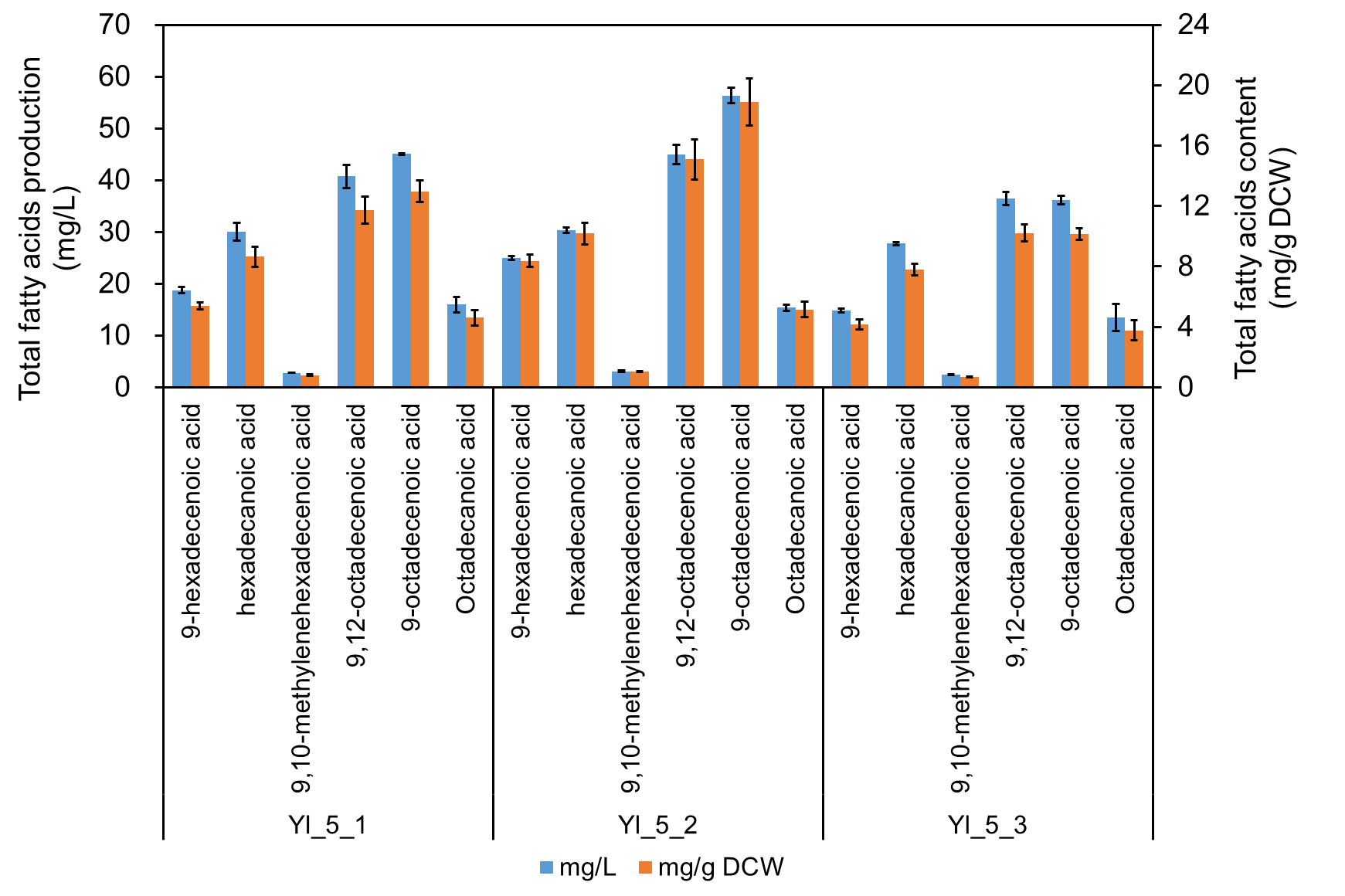
**

**Figure S14：The total contents of various fatty acids in each strain.** There are six kinds of fatty acids that has been obviously detected in the strains and their contents are quite different between the selected strains. All error bars indicate ±Standard Deviation, n=3.

**
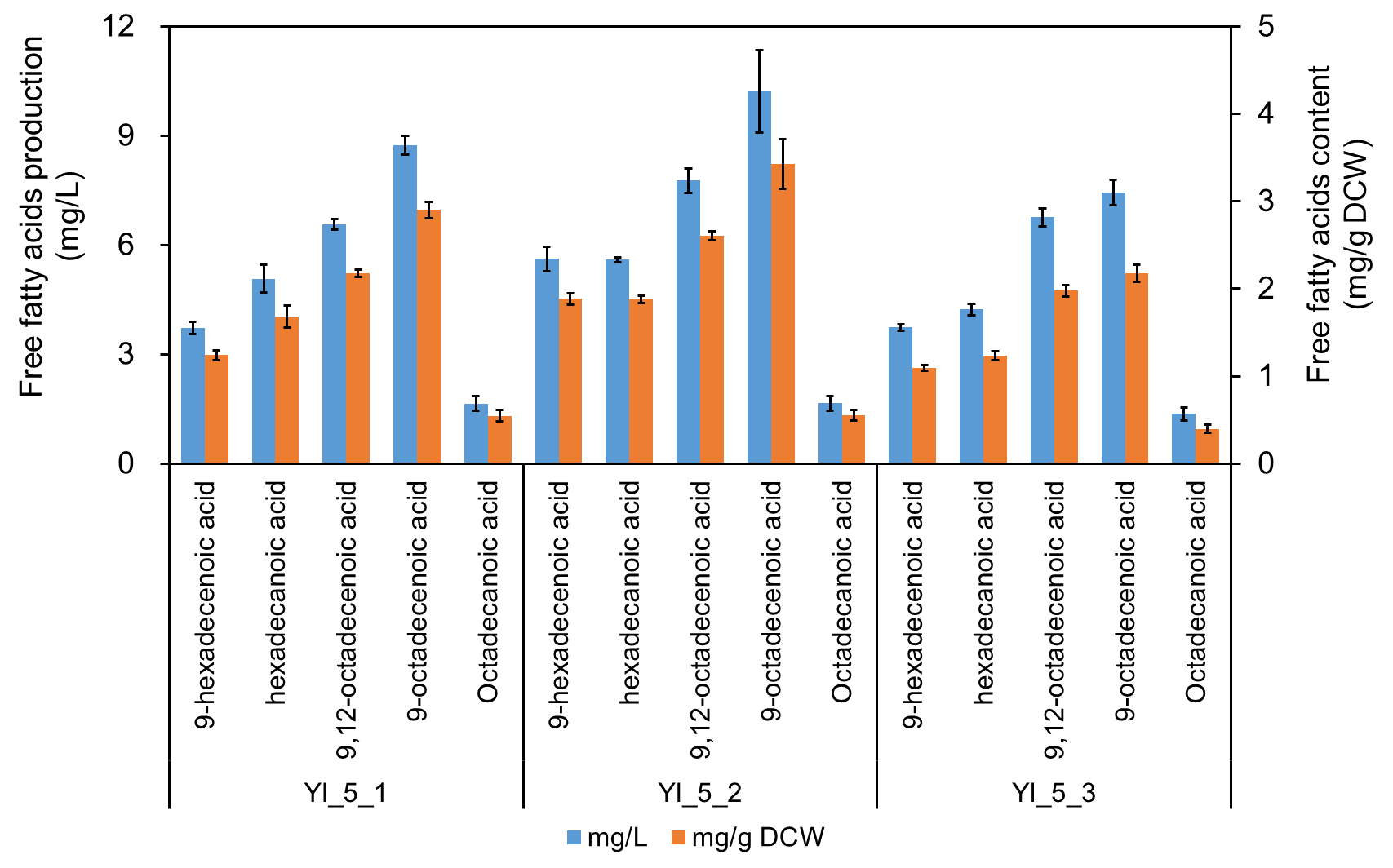
**

**Figure S15：The contents of various free fatty acids in each strain.** There are five kinds of free fatty acids that has been obviously detected in the strains and their contents are quite different between the selected strains. All error bars indicate ±Standard Deviation, n=3.

**
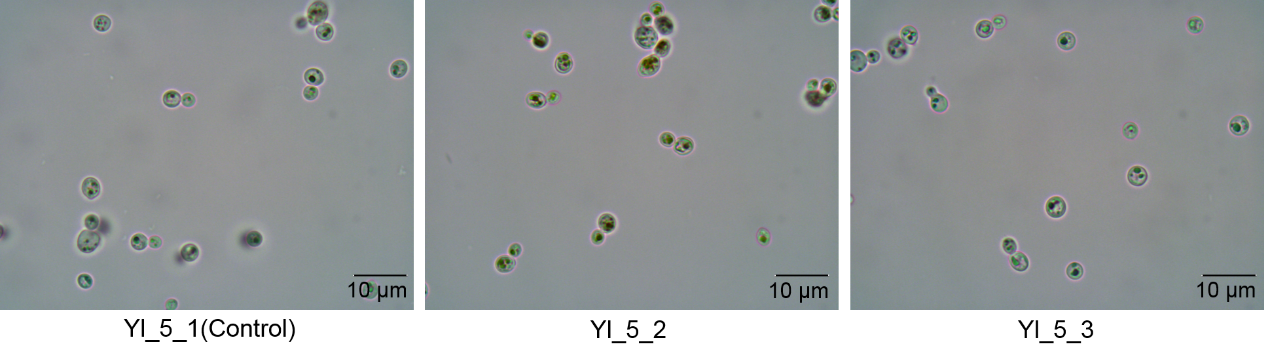
**

**Figure S16:** **Photomicrograph of lipid bodies after stained with sudan black B.** Lipid bodies were observed under microscopy after stained with sudan black B and appeared dark. The photos showed that the Yl_5_2 strain contained more droplets of lipid bodies than Yl_5_1 as control and Yl_5_3. Cell staining method: The cells were collected after centrifugation and stained with sudan black B for 90 seconds, then the cells were washed with 70% ethanol for twice and suspended with sterile water.
