# Supplementary material for: Engineering global transcription to tune lipophilic properties in Yarrowia lipolytica
Source: Biotechnol Biofuels. 2018 Apr 19;11:115. doi: 10.1186/s13068-018-1114-z (PMC5907459; doi:10.1186/s13068-018-1114-z)
Supplement: Supplementary file 2 — Additional file 2. Supporting Online Texts. [file 13068_2018_1114_MOESM2_ESM.docx]

**Additional file 2: Supporting Online Texts**

a) DNA sequence of the wild-type *Yl-SPT15*

1 atggatgccc tctctgcccc caccaaccct gcacaggcac agcaattcgt gacccagaac

61 aatctctcgt tccctcagga tgccgaggcc gacaccagtc tgattggaga ggtcaagcga

121 gagcaggtcg actctggtgt ctccggtatc gtccccacac tacagaacat tgttgcgacc

181 gtcaacctgg attgtcggct tgatctgaaa accattgcat tgcatgctcg aaatgccgag

241 tacaacccca agcgtttcgc tgccgtcatt atgcgtattc gagagcccaa gaccaccgct

301 ttgatcttcg catccggaaa gatggtcgta actggtgccc gaagcgagga tgactccaag

361 ctggccagcc ggaagtacgc ccgaatcatt cagaaacttg gattcaatgc caagttcact

421 gacttcaaaa tccagaacat tgtcggttcg tgcgatgtca agttccctat ccgacttgag

481 ggtcttgcat tttcacacgg tactttctcg tcgtacgagc ctgagttgtt ccctggtctc

541 atctaccgaa tggttaagcc caagattgtg ttgctgatct tcgtgtcggg taagattgtg

601 ctgactggtg ccaagcagcg agaggaaatt tacgcggcct tcgaagccat ctaccctgtg

661 ttgaacgagt ttagaaaggg gtag

b) DNA sequence of the mutant in Yl_5_2-module 1 (The mutant bases are in red)

1 atggatgccc tctctgcccc caccaaccct gcacaggcac agcaattcgt gacccagaac

61 aatctctcgt tccctcagga tgccgaggcc gacaccagtc tgattggaga ggtcaagcga

121 gagcaggtcg actctggtgt ctccggtatc gtccccacac tacagaacat tgttgcgacc

181 gtcaacctgg attgtcggct tgatctgaaa accattgcat tgcatgctcg aaatgccgag

241 tacaacccca agcgtttcgc tgccgtcatt atgcgtattc gagagcccaa gaccaccgct

301 ttgatcttcg catccggaaa gatggtcgta actggtgctc gaagcgagga tgactccaag

361 ctggccagcc ggaagtacgc ccgaatcatt cagaaacttg gattcaatgc caagttcact

421 gacttcaaaa tccagaacat tgtcggttcg tgcgatgtca agttccctat ccgacttgag

481 ggtcttgcat tttcacacgg tactttctcg tcgtacgagc ctgagttgtt ccctggtctc

541 atctaccgaa tggttaagcc caagattgtg ttgctgatct tcgtgtcggg taagattgtg

601 ctgactggtg ccaagcagcg agcggaaatt tacgcggcct tcgaagccat ctaccctgtg

661 ttgaacgagt ttagaaaggg gtag

c) DNA sequence of the mutant in Yl_5_2-module 5 (The mutant bases are in red)

1 atggatgccc tctctgcccc caccaaccct gcacaggcac agcaattcgt gacccagaac

61 aatctctcgt tcactcagga tgccgaggcc gacaccagtc tgattggaga ggtcaagcga

121 gagcaggtcg actctggtgt ctccggtatc gtccccacac tacagaacat tgttgcgacc

181 gtcaacctgg attgtcggct tgatctgaaa accattgcat tgcatgctcg aaatgccgag

241 tacaacccca agcgtttcgc tgccgtcatt atgcgtattc gagagcccaa gaccaccgct

301 ttgatcttcg catccggaaa gatggtcgta actggtgccc gaagcgagga tgactccaag

361 ctggccagcc ggaagtacgc ccgaatcatt cagaaacttg gattcaatgc caagttcact

421 gacttcaaaa tccagaacat tgtcggttcg tgcgatgtca agttccctat ccgacttgag

481 ggtcttgcat tttcacacgg tactttctcg tcgtacgagc ctgagttgtt ccctggtctc

541 atctaccgaa tggttaagcc caagattgtg ttgctgatct tcgtgtcggg taagattgtg

601 ctgactggtg ccaagcagcg agaggaaatt tacgcgacct tcgaagtcat ctaccctgtg

661 ttgaacgagt ttagaaaggg gtag

d) DNA sequence of the mutant in Yl_5_2-module 2 (The mutant bases are in red)

1 atggatgccc tctctgcccc caccaaccct gcacaggcac agcaattcgt gacccagaac

61 aatctctcgt tccctcagga tgccgaggcc gacaccagtc tgattggaga ggtcaagcga

121 gagcaggtcg actctggtgt ctccggtatc gtccccacac tacagaacat tgttgcgacc

181 gtcaacctgg attgtcggct tgatctgaaa accattgcat tgcatgctcg aaatgccgag

241 tacaacccca agcgtttcgc tgccgtcatt atgcgtattc gagagcccaa gaccaccgct

301 ttgatcttcg catccggaaa gatggtcgta actggtgccc gaagcgagga tgactccaag

361 ctggccagcc ggaagtacgc ccgaatcatt cagaaacttg gattcaatgc caagttcact

421 gacttcaaaa tccagaacat tgtcggttcg tgcgatgtca agttccctat ccgacttgag

481 ggtcttgcat tttcacacgg tactttctcg tcgtacgagc ctgagttgtt ccctggtctc

541 atctaccgaa tggttaagcc caagattgtg ttgctgatct tagtgtcggg taagattgtg

601 ctgactggtg ccaagcagcg agaggaaatt tacgcggcct tcgaagccat ctaccctgtg

661 ttgaacgagt ttagaaaggg gtag
