# Supplementary material for: Engineering global transcription to tune lipophilic properties in Yarrowia lipolytica
Source: Biotechnol Biofuels. 2018 Apr 19;11:115. doi: 10.1186/s13068-018-1114-z (PMC5907459; doi:10.1186/s13068-018-1114-z)
Supplement: Supplementary file 3 — Additional file 3: Table S1. Oligonucleotide primers used for Q-PCR. Table S2. Primers for PCR amplification to identify the genotypes of mutants. Table S3. Primers for PCR amplification to identify the genotypes of mutants and the insertion site. Table S4. Up-regulated genes in the pathways of ketone bodies and alpha-linolenic acid metabolism in Yl_5_2. Table S5. Down-regulated genes in the pathway of fatty acid biosynthesis in Yl_5_2. Table S6. Down-regulated genes in the pathway of fatty acid degradation in Yl_5_3. Table S7. All strains can be obtained according to their standard numbers in laboratory. [file 13068_2018_1114_MOESM3_ESM.docx]

**Additional file 3: Table S1-S7**

**Table S1: Oligonucleotide primers used for Q-PCR**

| Gene | Primers | Sequence(5'-3') | Tm | GC% |
| --- | --- | --- | --- | --- |
| ACT(YALI0D08272g) | Yarrowia-ACT-F | CTCCATCAAGGTCAAGAT | 47.9 | 44.4 |
|  | Yarrowia-ACT-R | TACCAAGAGAAGCAAGAA | 46.6 | 38.9 |
| EXP1p | EXP1p-F | CTTGCTTGCGAACCTAAT | 49.7 | 44.4 |
|  | EXP1p-R | TAATTGGATTGCCTGATGG | 49.2 | 42.1 |
| TEFp | TEFp-F | GACTTTAGCCAAGGGTATA | 47.6 | 42.1 |
|  | TEFp-R | GTGAGTTGACAAGGAGAG | 49.4 | 50.0 |
| GPDp | GPDp-F | TCCACAGAGTAGAGTCAA | 48.4 | 44.4 |
|  | GPDp-R | AATAGAGCGGGAATATAACC | 48.2 | 40.0 |
| GPATp | GPATp-F | GGTGCTTGTATGGATAGAGAA | 51.1 | 42.9 |
|  | GPATp-R | GGAGGTGTATTGATGGTGAA | 51.5 | 45.0 |
| GUT2(YALI0B13970g) | GUT2-F | CGCCATTGTCTACTATGATG | 50.1 | 45.0 |
|  | GUT2-R | CCTTCTCAACAGCAGTCA | 51.7 | 50.0 |
| Yl-SPT15  (YALIOB23056g) | YI-SPT15-F | TGGTCTCATCTACCGAAT | 48.9 | 44.4 |
|  | YI-SPT15-R | CCAGTCAGCACAATCTTA | 48.4 | 44.4 |

**Table S2:** **Primers for PCR amplification to identify the genotypes.**

| Primer | Sequence 5’ to 3’ |
| --- | --- |
| H0-F | AGTGCTTTTAACTAAGAATTATTAGTCTTT |
| H0-R | AAAGCCACGCGTGTGCACCTTTTTTTTCCC |
| H1-F | GTCTGAAGAATGAATGATTTGATGATTTCT |
| H1-R | TAATAATACTCCTCAAATTGCTACCACGAC |
| H2-F | GTGAATTTACTTTAAATCTTGCATTTAAAT |
| H2-R | ATCCACAATGTATCAGGTATCTACTACAGA |
| H3-F | GTTAATTCAAATTAATTGATATAGTTTTTT |
| H3-R | AAAGATGAGCTAGGCTTTTGTAAAAATATC |
| H4-F | ATTGAATTGAATTGAAATCGATAGATCAAT |
| H4-R | AAAACTGCATAAAGGCATTAAAAGAGGAGC |
| H5-F | AAATAAGGAGATTGATAAGACTTTTCTAGT |
| H5-R | GAATCGGACTGGGATAAATCAACATTTGGA |
| H6-F | GATTAATATAATTATATAAAAATATTATCT |
| H6-R | TCTTTTTATTAGAAAAAGCGCCTTGCTTTT |
| H7-F | ACAAATCGCTCTTAAATATATACCTAAAGA |
| H7-R | TATTAGTGCACATAATGTAGTTACTTGGAC |
| H8-F | GAGTAATAATTATTGCTTCCATATAATATT |
| H8-R | TCTGTCTTCTCCTTCTCTTACAAAAAAACC |

**Table S3:** **Primers for PCR amplification to identify the genotypes of mutants and the insertion site.**

| Primer | Sequence 5’ to 3’ |
| --- | --- |
| Module 1-F | CGCCACCCTTTGGTTCTTC |
| Module 1-R | ATAACCCATGTGTGTGTTTCCA |
| Module 2-F | GGCCTTTGATTCTTCCCTACA |
| Module 2-R | CTCTCCGAGCAGTATGAGGCTCT |
| Module 3-F | AGAGCACCAACCCACTATCCAA |
| Module 3-R | AATTGTGCACACAGAACCGG |
| Module 4-F | AGTCGCTAGCAACACACACTCTCT |
| Module 4-R | GAGGGCGTGAATGTAAGCGT |
| Module 5-F | ACACACCACATCACACATACAACCAC |
| Module 5-R | TGCAATCAATACATAATCTTTATTCATACAAT |
| GUT2L HR-F | GAGTCAGTAGCAACGTAGACTTTG |
| GUT2L HR-R | GTCAGTCTTGGTCTTCTTAGTTTC |
| GUT2R HR-F | TGCTAAGCGCATGTGATAA |
| GUT2R HR-R | AACAACACCGTTGAGCTGG |

**Table S4:** **Up-regulated genes in the pathways of ketone bodies and alpha-linolenic acid metabolism in Yl_5_2.**

| Term | Gene | Gene-name | log2  (change) | p-value | Function |
| --- | --- | --- | --- | --- | --- |
| Synthesis and degradation of ketone bodies | *YALI0F30481g* | *ERG13* | 0.67969 | 2.53E-21 | hydroxymethylglutaryl-CoA synthase |
|  | *YALI0B22550g* | *HMGL* | 0.59017 | 2.17E-15 | hydroxymethylglutaryl-CoA lyase |
|  | *YALI0E11099g* | *ERG10* | 0.39309 | 1.96E-07 | acetyl-CoA C-acetyltransferase |
|  | *YALI0F26587g* | *OXCT* | 0.3726 | 6.11E-04 | 3-oxoacid CoA-transferase |
| alpha-Linolenic acid metabolism | *YALI0F10857g* | *ACX* | 0.37429 | 1.19E-10 | peroxisomal acyl-coenzyme A oxidase |
|  | *YALI0E18568g* | *POT1* | 0.31147 | 1.01E-07 | acetyl-CoA C-acyltransferase |
|  | *YALI0D24750g* | *ACX* | 0.20529 | 4.43E-03 | peroxisomal acyl-coenzyme A oxidase |
|  | *YALI0F10010g* | *TGL4* | 0.18157 | 1.33E-03 | Multifunctional lipase/hydrolase/phospholipase; |

**Table S5: Down-regulated genes in the pathway of fatty acid biosynthesis in Yl_5_2**

| Gene | Gene-name | log2(change) | p-value | Function |
| --- | --- | --- | --- | --- |
| *YALI0B15059g* | *FAS1* | -0.63187 | 3.61E-07 | Beta subunit of fatty acid synthetase; complex catalyzes the synthesis of long-chain saturated fatty acids |
| *YALI0C11407g* | *ACC1* | -0.36848 | 2.02E-03 | Acetyl-CoA carboxylase, biotin containing enzyme |
| *YALI0B19382g* | *FAS2* | -0.33108 | 1.71E-02 | Alpha subunit of fatty acid synthetase; complex catalyzes the synthesis of long-chain saturated fatty acids |
| *YALI0D17864g* | *FAA* | -0.1511 | 9.25E-03 | Medium and long chain fatty acyl-CoA synthetase |

**Table S6: Down-regulated genes in the pathway of fatty acid degradation in Yl_5_3**

| Gene | Gene-name | log2(change) | p-value | Function |
| --- | --- | --- | --- | --- |
| *YALI0A16379g* | *ADH5* | -2.675 | 1.51E-90 | Alcohol dehydrogenase isoenzyme V |
| *YALI0B10406g* | *ECH* | -1.8995 | 6.92E-77 | enyol-CoA hydratase |
| *YALI0A15147g* | *ADH5* | -1.55 | 1.53E-99 | Alcohol dehydrogenase isoenzyme V |
| *YALI0E11099g* | *ERG10* | -0.82676 | 7.37E-13 | acetyl-CoA C-acetyltransferase |
| *YALI0D24750g* | *ACX* | -0.69235 | 8.18E-12 | peroxisomal acyl-coenzyme A oxidase |
| *YALI0E00264g* | *HFD1* | -0.65141 | 4.58E-26 | Dehydrogenase involved in ubiquinone and sphingolipid metabolism |
| *YALI0F10857g* | *ACX* | -0.33566 | 2.61E-07 | peroxisomal acyl-coenzyme A oxidase |
| *YALI0F23749g* | *ACDH* | -0.32806 | 2.52E-03 | acetaldehyde dehydrogenase |
| *YALI0B01298g* | *ALD5* | -0.29572 | 1.23E-09 | Mitochondrial aldehyde dehydrogenase; involved in regulation or biosynthesis of electron transport chain components and acetate formation |
| *YALI0E18568g* | *POT1* | -0.24103 | 2.42E-04 | acetyl-CoA C-acyltransferase |
| *YALI0F23793g* | *ALD4* | -0.20649 | 1.39E-05 | Mitochondrial aldehyde dehydrogenase; required for growth on ethanol and conversion of acetaldehyde to acetate |
| *YALI0B08536g* | *ERG10* | -0.16877 | 8.90E-03 | acetyl-CoA C-acetyltransferase |
| *YALI0F09603g* | *ADH5* | -0.16618 | 1.74E-03 | Alcohol dehydrogenase isoenzyme V |
| *YALI0D17864g* | *FAA* | -0.14917 | 1.82E-03 | Medium and long chain fatty acyl-CoA synthetas |

**Table S7: All strains can be obtained according to their standard numbers in laboratory.**

| Strain in the article | Standard number in laboratory | Plasmid contained |
| --- | --- | --- |
| Yl_5_1 | SyBE_YL03060001 |  |
| Yl_5_2 | SyBE_YL03060002 |  |
| Yl_5_3 | SyBE_YL03060003 |  |
| Yl_5_2_0 | SyBE_YL03060004 | pLD-EcYl |
| Yl_5_2_1 | SyBE_YL03060005 | pLD-EcYl-1 |
| Yl_5_2_2 | SyBE_YL03060006 | pLD-EcYl-2 |
| Yl_5_2_3 | SyBE_YL03060007 | pLD-EcYl-3 |
| Yl_5_2_4 | SyBE_YL03060008 | pLD-EcYl-4 |
| Yl_5_2_5 | SyBE_YL03060009 | pLD-EcYl-5 |
| Yl_5_2_6 | SyBE_YL030600010 | pLD-EcYl-6 |
| Yl_5_2_7 | SyBE_YL030600011 | pLD-EcYl-7 |
| Yl-ini | SyBE_YL030600012 |  |
