# Supplementary figures and images for: Engineering global transcription to tune lipophilic properties in Yarrowia lipolytica
Source: Biotechnol Biofuels. 2018 Apr 19;11:115. doi: 10.1186/s13068-018-1114-z (PMC5907459; doi:10.1186/s13068-018-1114-z)

Cluster analysis of differentially expressed genes

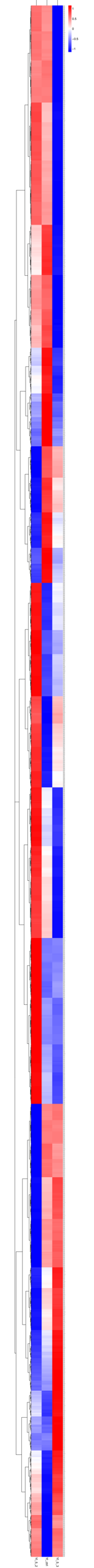

Supplement: Supplementary file 4 — Additional file 4.Transcription heat map. The differential expressed genes are listed (p < 0.05). [file 13068_2018_1114_MOESM4_ESM.pdf]
